# Mind–Body Therapies for Multimorbidity: A Bibliometric Analysis and Evidence Map

**DOI:** 10.3390/healthcare14182902

**Published:** 2026-09-08

**Authors:** Xia Li, Heng Yin, Zhiqiang Li, Yiying Wang, Yi Yuan, Ran Chen, Jun Fang, Jianping Liu

**Affiliations:** 1 Centre for Evidence-Based Chinese Medicine, Beijing University of Chinese Medicine, Beijing 100029, China; 20240941074@bucm.edu.cn (X.L.); 20200621018@bucm.edu.cn (H.Y.); lizhiqiang@bucm.edu.cn (Z.L.); 20240941073@bucm.edu.cn (Y.W.); yiyuan@bucm.edu.cn (Y.Y.); 2 School of Chinese Medicine, Beijing University of Chinese Medicine, Beijing 100029, China; 20230941042@bucm.edu.cn; 3 Inner Mongolia Traditional Chinese and Mongolian Medical Research Institute, Hohhot 010010, China; 340695804qq@gmail.com; 4 Center for Evidence-Based Medicine, Hospital of Chengdu University of Traditional Chinese Medicine, Chengdu 610072, China

**Keywords:** multimorbidity, mind-body therapies, mindfulness, bibliometric analysis, evidence mapping

## Abstract

**Objectives:** Multimorbidity is a growing global health challenge, yet the role of mind–body therapies in addressing its complex health burdens remains insufficiently characterised. This study aimed to map the research landscape and clinical evidence structure of mind–body therapies for multimorbidity. **Methods:** Bibliometric analysis integrated with evidence mapping was conducted using English-language publications indexed in the Web of Science Core Collection, PubMed, and Embase from January 2004 to July 2026. CiteSpace and R were used to construct knowledge networks, visualise temporal, geographical, collaborative, and thematic patterns, and generate evidence maps. Clinical studies were coded according to intervention type, comparator, comorbidity pattern, outcome domain, and reported outcome direction. **Results:** Of 6441 records identified, 119 publications were retained for bibliometric analysis and 77 clinical studies for evidence mapping. Publication activity increased after 2017 but remained geographically concentrated, with limited international collaboration. Keyword, clustering, timeline, and co-citation analyses showed sustained research attention to chronic pain, post-traumatic stress disorder, substance use disorders, anxiety disorders, mindfulness-based approaches, and biofeedback or neurofeedback. The evidence map covered 53 condition combinations, most of which were represented by only one clinical study. Mindfulness-based interventions were the most frequently studied approach (31.2%), followed by multicomponent mind–body interventions. Randomised controlled designs represented only a minority of the clinical evidence, and many studies had no comparator. Psychological outcomes and clinical symptoms were commonly assessed, although outcome categories were not mutually exclusive and findings varied across studies. **Conclusions:** Research on mind–body therapies for multimorbidity is expanding, but the evidence remains heterogeneous across condition combinations, interventions, comparators, and outcomes. Publication frequency and bibliometric prominence do not establish comparative effectiveness. Future studies should include broader condition combinations, use robust comparative designs, and report standardised outcomes and safety data.

## 1. Introduction

Multimorbidity, commonly defined as the coexistence of two or more chronic conditions within a single individual, has become one of the most pressing challenges facing global public health. Its prevalence has increased substantially across diverse populations, driven by population ageing, lifestyle transitions, and advances in diagnostic capacity. A recent large-scale systematic review and meta-analysis encompassing more than 15 million individuals across 54 countries estimated the global prevalence of multimorbidity among adults at approximately 37.2%, with more than half of individuals aged 60 years and above affected [1]. Beyond its high prevalence, multimorbidity is associated with a range of adverse outcomes, including elevated mortality risk, progressive functional decline, reduced quality of life, and substantial healthcare utilisation and costs [2,3]. The burden of multimorbidity is not evenly distributed, however, with marked disparities across regions, socioeconomic strata, and healthcare systems, further complicating clinical management and policy responses [4]. Collectively, these findings underscore the growing, heterogeneous, and increasingly unmanageable burden that multimorbidity places on healthcare systems worldwide.

Critically, multimorbidity is not merely the concurrent presence of multiple diseases, but involves a complex and dynamic interplay among interacting conditions, symptoms, and functional impairments that collectively exceed the sum of their parts [2]. Individuals with multimorbidity often present with overlapping and mutually reinforcing symptom clusters, encompassing chronic pain, anxiety, depression, sleep disturbance, and reduced physical functioning [5,6]. These multidimensional and interconnected health challenges introduce substantial clinical complexity, requiring concurrent management of multiple conditions and often involving polypharmacy, increased treatment burden, and potentially conflicting recommendations from disease-specific clinical guidelines. Such difficulties expose fundamental limitations of conventional single-disease-oriented healthcare models and underscore the need for more integrated, complexity-sensitive, and person-centred approaches. In this context, the multidimensional and mutually reinforcing nature of multimorbidity calls for integrative intervention strategies capable of simultaneously addressing physical and psychological dimensions, rather than targeting individual conditions in isolation.

In response to these challenges, and as integrated healthcare models gain traction in global health policy, non-pharmacological and integrative approaches to chronic disease management have attracted growing attention [7]. Among these, mind–body therapies have become increasingly prominent. According to the National Centre for Complementary and Integrative Health (NCCIH), mind–body practices encompass a range of interventions, including meditation, mindfulness-based programmes, yoga, tai chi, relaxation techniques, and biofeedback, which are designed to influence health through brain–body interactions [8]. As a class of non-pharmacological interventions, mind–body therapies distinctively integrate cognitive, emotional, and behavioural processes with physiological regulation, thereby addressing health through multiple interacting pathways simultaneously. A growing body of evidence indicates that these therapies can produce beneficial effects across multiple domains, including psychological well-being, physical symptoms, and functional outcomes such as sleep quality and health-related quality of life. These multidomain effects are thought to be mediated through mechanisms such as modulation of the autonomic nervous system, attenuation of hypothalamic–pituitary–adrenal (HPA) axis reactivity, reduction in systemic stress responses, and enhancement of emotional regulation and self-regulatory capacity. Importantly, the holistic and integrative nature of mind–body therapies aligns closely with the complex, interconnected health needs of individuals living with multimorbidity. By targeting shared symptom clusters and the underlying biopsychosocial mechanisms that cut across multiple conditions, these approaches offer a theoretically coherent and clinically promising pathway toward advancing whole-person, patient-centred care.

Previous bibliometric studies have examined mind–body therapies broadly or investigated multimorbidity as a separate research field, but they have not described how intervention categories, diagnosed condition combinations, comparators, and outcome domains are connected within the clinical literature at their intersection [9,10]. Bibliometric analysis provides a quantitative approach to characterising publication, collaboration, co-citation, and thematic patterns [11], while evidence mapping describes the distribution of the clinical literature across predefined categories. The principal contribution of the present study is therefore the integration of bibliometric analysis with a publication-level clinical evidence map. We aimed (1) to describe the temporal, geographic, collaborative, and thematic patterns of English-language publications on mind–body therapies for comorbidity and multimorbidity and (2) to map the included clinical publications by intervention type, comparator, diagnosed condition combination, outcome domain, and reported outcome direction. Because no effect sizes, risk-of-bias assessments, or certainty ratings were synthesised, this study was designed to characterise research patterns and the distribution of clinical evidence rather than to estimate intervention effects.

## 2. Materials and Methods

This study was conducted and reported in accordance with the Preliminary Guideline for Reporting Bibliometric Reviews of the Biomedical Literature (BIBLIO) [12]. Supplementary Table S1 provides an item-by-item checklist showing where each applicable guideline item is addressed; the main procedures are also reported below.

### 2.1. Data Sources and Search Strategy

Bibliographic records were retrieved from the Science Citation Index Expanded in the Web of Science Core Collection (WoSCC), PubMed, and Embase. Search terms for mind–body therapies were combined with terms for comorbidity and multimorbidity using the Boolean operator “AND.” Searches covered publications from January 2004 through July 2026 and were limited to English-language articles and reviews. Supplementary Table S2 provides the full search strategy for each database, including field tags, date limits, and language and publication-type filters.

### 2.2. Inclusion and Exclusion Criteria

Publications were eligible for bibliometric analysis if they (1) examined mind–body therapies in populations with comorbidity or multimorbidity, (2) were written in English, and (3) were classified as articles or reviews. In this study, multimorbidity was defined as the coexistence of two or more formally diagnosed conditions, without designating an index condition. A formally diagnosed mental disorder was considered an eligible condition, whereas an elevated symptom score alone was not. The scope of eligible mind–body therapies was based on definitions provided by the NCCIH and relevant Cochrane reviews [13].

Publications were excluded if they (1) included only one diagnosed condition accompanied by anxiety, depression, fatigue, stress, sleep problems, or similar symptom-level manifestations, without a second formally diagnosed condition; (2) mentioned comorbidity or multimorbidity only incidentally; (3) evaluated a solely psychological intervention without an eligible mind–body component; or (4) were duplicate or retracted records. A formally diagnosed mental disorder counted as an eligible condition, whereas an elevated symptom score alone did not.

After title and abstract screening, potentially eligible records were assessed in full text for inclusion in the evidence map. We included primary intervention publications and secondary analyses that evaluated an eligible mind–body intervention in participants with multiple coexisting diagnosed conditions and reported at least one intervention-related clinical outcome or treatment-effect estimate. Secondary analyses were eligible when they provided independent intervention-related findings. Publications without clinical intervention data were excluded.

Two reviewers independently screened all records by title and abstract. Full texts were consulted only when eligibility could not be determined from the available information. Disagreements were resolved through discussion, with a third reviewer consulted when necessary. Inter-reviewer agreement for title and abstract screening was assessed using Cohen’s kappa.

### 2.3. Bibliometric Analysis

Bibliometric analyses were conducted in CiteSpace (version 6.4.R1; Drexel University, Philadelphia, PA, USA), R (version 4.3.2; R Foundation for Statistical Computing, Vienna, Austria), and WPS Office Spreadsheets (version 7.5.1; Kingsoft Office Software Corporation, Zhuhai, China). Before analysis, the bibliographic records were cleaned and standardised.

CiteSpace was used to construct and visualise networks of author collaboration, institutional collaboration, keyword co-occurrence, and reference co-citation [14,15]. The analysis spanned 2006 to 2026 in one-year slices. For the keyword co-occurrence network, author keywords were used as the term source, and the 50 highest-ranked nodes were retained in each slice under the Top N criterion. A separate keyword network was generated for clustering and timeline visualisation using the g-index. The co-citation network used cited references as the node type and the g-index for node selection. For all analyses using the g-index, the parameter k was set to 25. Within each slice, link strength was calculated using cosine similarity. The Pathfinder and Pruning Sliced Network algorithms were applied to simplify the networks.

Keyword clusters were labelled using LLR and evaluated using modularity Q and the weighted mean silhouette value. The timeline view was generated from the same cluster network. Keyword bursts were detected using Kleinberg’s algorithm as implemented in CiteSpace, with the default settings and no additional minimum-duration or other parameter constraints. In the networks, node size represents publication count, keyword frequency, or co-citation frequency, as applicable. Links represent collaboration, keyword co-occurrence, or reference co-citation, and link thickness represents relationship strength. Betweenness centrality describes the extent to which a node bridges different parts of a network; it was not interpreted as a direct measure of research quality, academic influence, or clinical importance.

R was used to process data and to analyse and visualise annual publication trends, publication distributions by country or region, and evidence-map results. WPS Office Spreadsheets was used for data management, descriptive analyses, and verification of results.

### 2.4. Data Extraction and Coding for the Evidence Map

For the evidence map, one reviewer extracted and coded study design, intervention, comparator, diagnosed conditions, condition combinations, outcomes, and outcome findings using a standardised form. A second reviewer independently checked a random sample of included studies for completeness and consistency. Disagreements were resolved through discussion or consultation with a third reviewer. The coding framework and extracted study-level data are provided in Supplementary Tables S3 and S4.

Conditions were classified according to the WHO ICD-11 framework, and condition combinations were treated as nondirectional. The treemap used the following four mutually exclusive categories: mental–mental, mental–physical, physical–physical, and unspecified multimorbidity. The Sankey diagram used more detailed ICD-11 disease-system categories. Intervention types were mutually exclusive and assigned according to the defining active component. Single modalities were coded in their specific categories. Multicomponent interventions included two or more eligible mind–body modalities, or an eligible modality combined with a structured physical or educational component when no single modality clearly predominated. A general mind–body category was used when the intervention was described too broadly for classification into a specific modality.

Outcomes were assigned to nine domains using a framework informed by the needs-based quality-of-life model for multimorbidity and the WHOQOL framework [16,17]. Each domain was counted once per study for study-level analyses. Outcome findings were coded as beneficial, non-significant, unfavourable, safe, unsafe, or unclear; no mixed category was used. For the Sankey diagram, each extracted outcome occurrence was retained, allowing one study to contribute multiple ribbons. Study-level percentages used all included studies as the denominator, whereas outcome nodes and ribbons were weighted by the number of extracted outcome occurrences. No meta-analysis, risk-of-bias assessment, or certainty-of-evidence assessment was conducted.

## 3. Results

### 3.1. Literature Search and Study Selection

**Figure 1:**
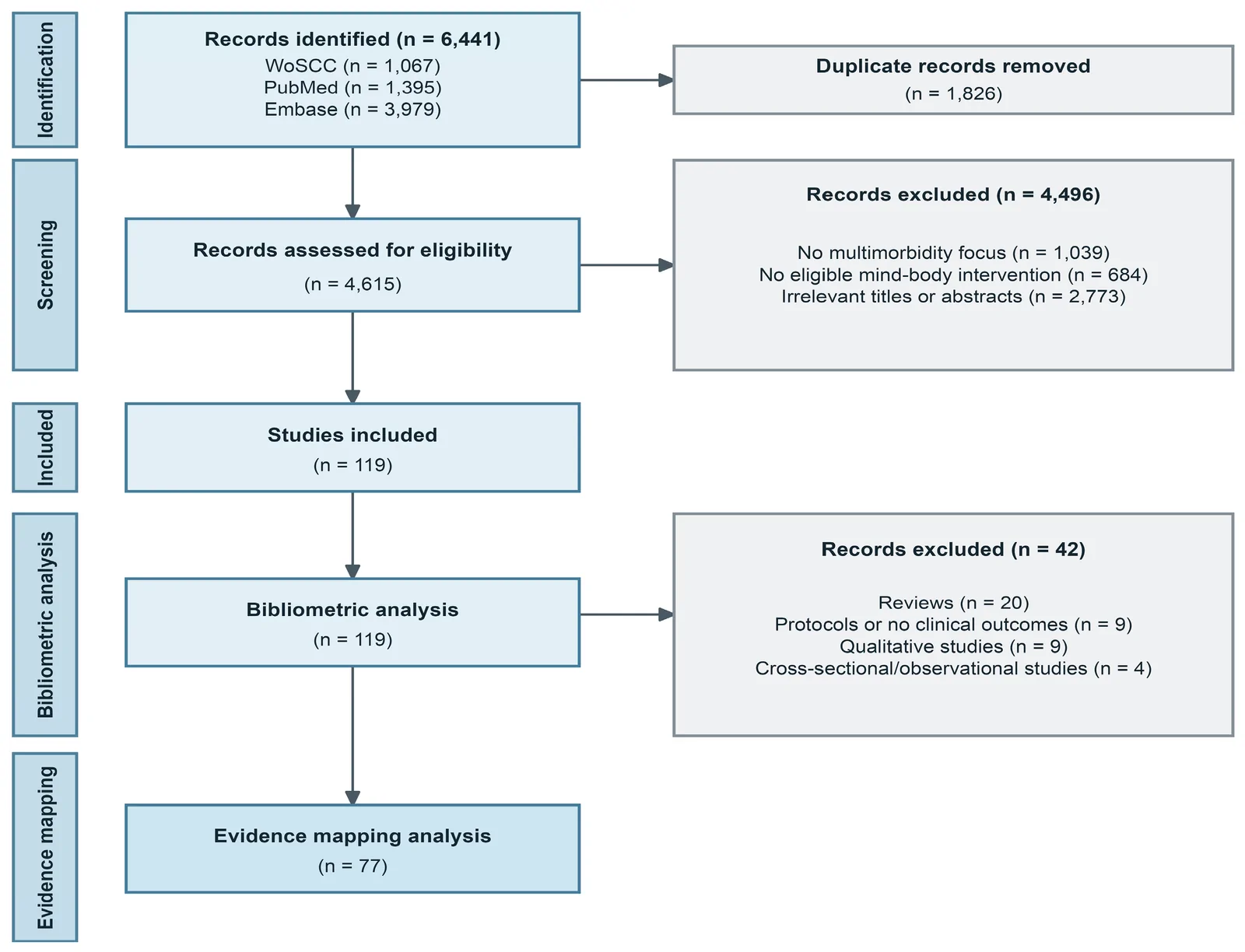
Flowchart of study selection for bibliometric analysis and evidence mapping.

The database searches retrieved 6441 records from WoSCC (*n* = 1067), PubMed (*n* = 1395), and Embase (*n* = 3979). After removal of 1826 duplicate records, 4615 records underwent title and abstract screening. The two reviewers identified 133 and 119 records as potentially eligible, respectively. Following discussion and consensus, 119 publications were retained for bibliometric analysis. Inter-reviewer agreement for title and abstract screening was high (Cohen’s k = 0.94). A further 42 publications were excluded from the evidence map: 20 reviews, 9 protocols or publications without reported clinical outcomes, 9 qualitative studies, and four cross-sectional or observational studies without an eligible intervention. The final evidence map included 77 clinical publications. Figure 1 presents the publication selection and evidence-map inclusion process.

### 3.2. Temporal Trends, Geographic Distribution, and International Collaboration

**Figure 2:**
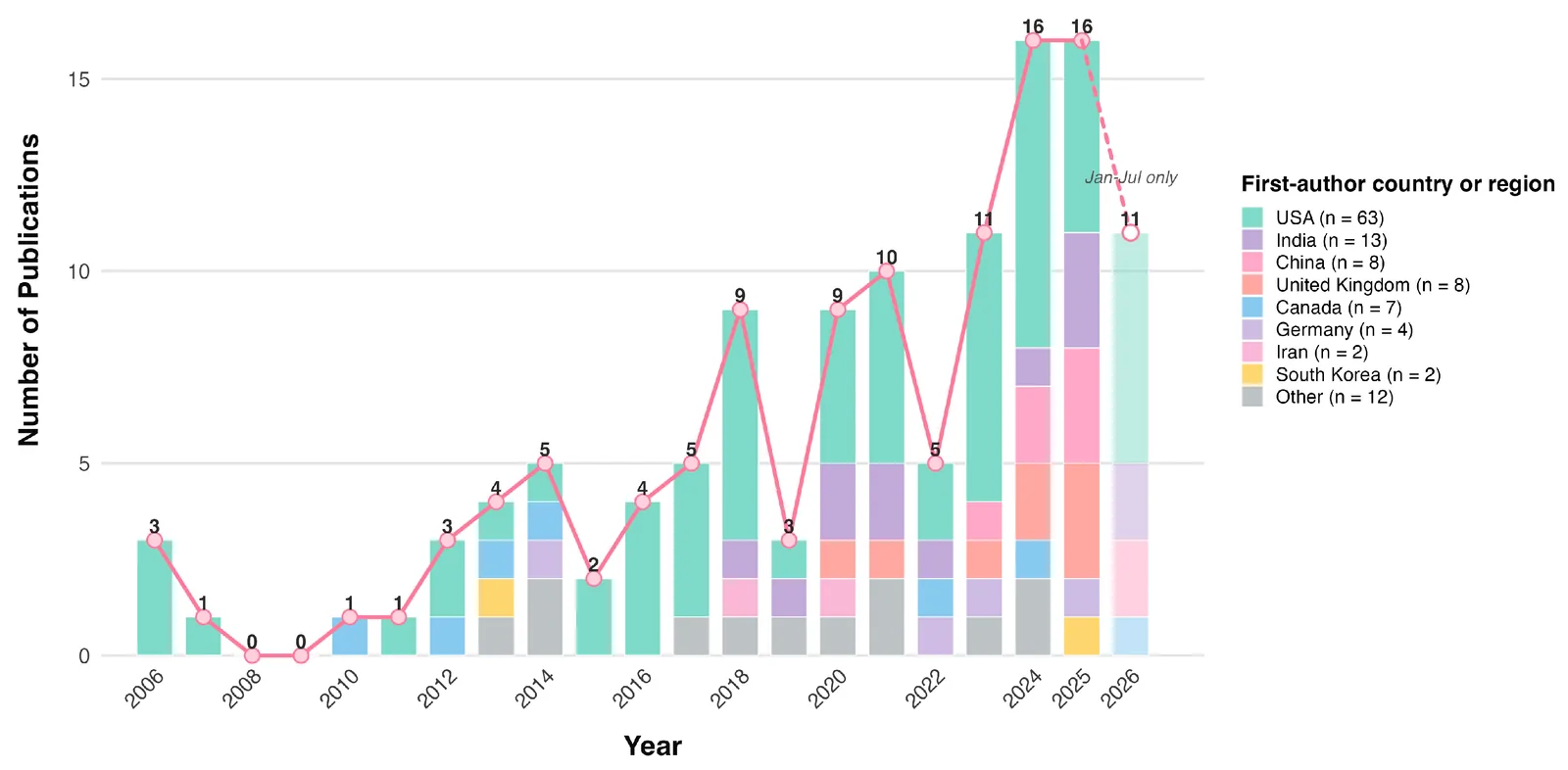
Annual publication trends in research on mind–body therapies for multimorbidity. Bars are stacked by first-author country or region, and the superimposed line represents annual publication totals. Countries or regions with one publication are grouped as Other. Data for 2026 cover January through July only.

Based 119 publications originated from 20 countries or regions. Figure 2 shows annual publication output by first-author country or region. Although the search covered January 2004 through July 2026, no eligible publications were found in 2004 or 2005; the first appeared in 2006. From 2006 through 2017, annual output remained low, at 0 to 5 publications per year. Despite year-to-year fluctuations, output later increased to 16 publications in both 2024 and 2025, the highest annual totals among complete years. The 11 publications indexed from January through July 2026 are shown separately and were excluded from the interpretation of temporal trends because 2026 was incomplete.

**Figure 3:**
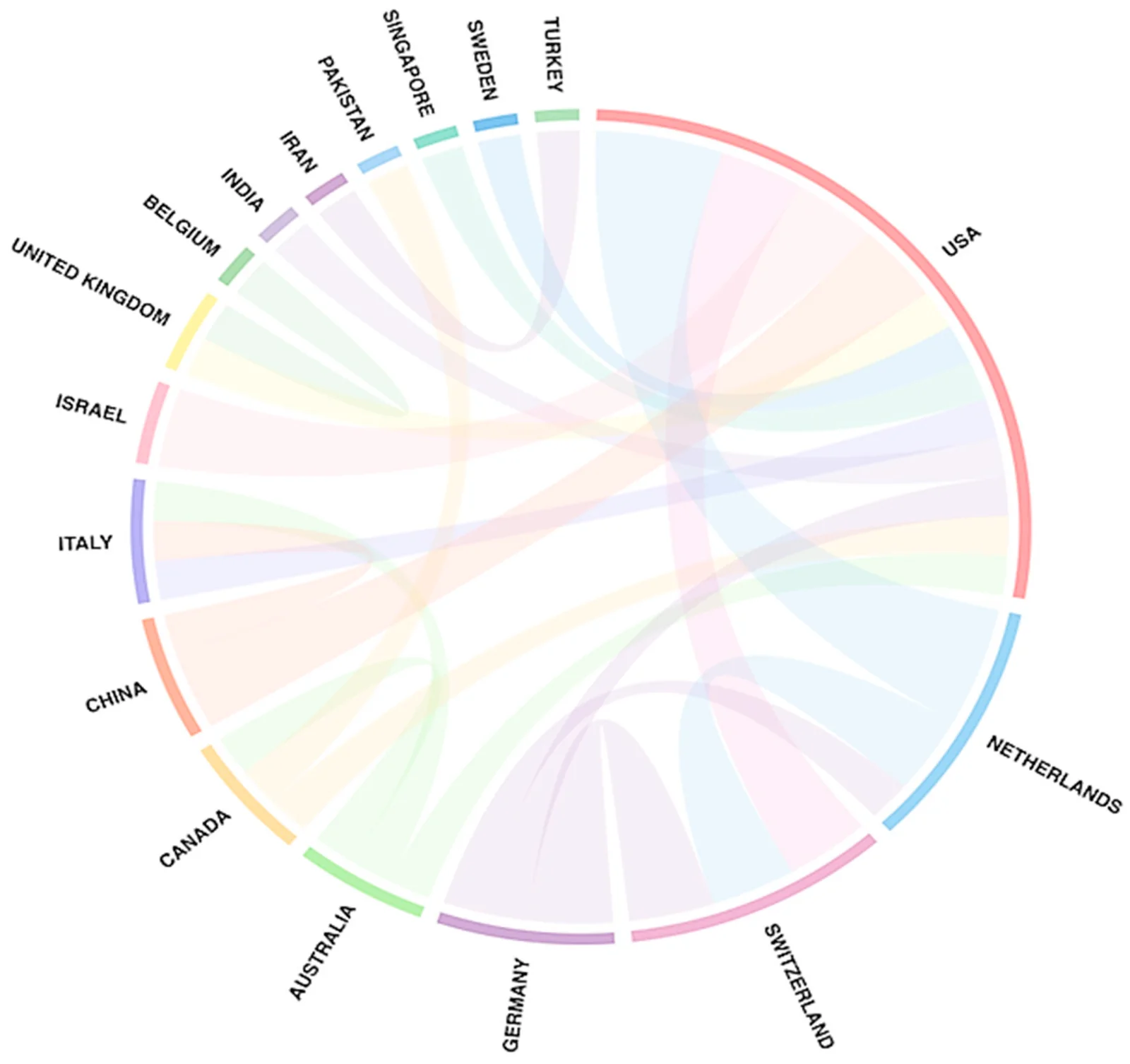
International collaboration network of countries in research on mind–body therapies for multimorbidity. Sectors represent countries involved in international collaboration. Ribbons connect collaborating country pairs, and ribbon width is proportional to the number of publications involving each pair. For publications involving more than two countries, each unique country pair was counted once.

The United States contributed 63 publications (52.94%), followed by India with 13 (10.92%). China and the United Kingdom each contributed eight (6.72%), Canada seven (5.88%), Germany four (3.36%), and South Korea and Iran two each (1.68%). The other 12 countries or regions contributed one publication each. We assessed international collaboration separately using all author affiliations. Eighteen publications (15.13%) included authors from more than one country. The collaboration network contained 17 countries and 21 unique bilateral links, representing 28 country-pair occurrences (Figure 3). The United States had links to 12 partner countries and appeared in 17 country-pair occurrences. The Netherlands–United States partnership was the most frequent, appearing in three publications. The China–United States, Germany–Switzerland, Israel–United States, Netherlands–Switzerland, and Switzerland–United States partnerships each appeared in two publications; the remaining 15 bilateral links each occurred once. International collaboration was therefore limited to a small proportion of the included publications and was concentrated in a few recurring partnerships. The network describes collaboration within this dataset and does not directly measure national influence or research quality.

### 3.3. Keyword Analysis and Clustering

**Figure 4:**
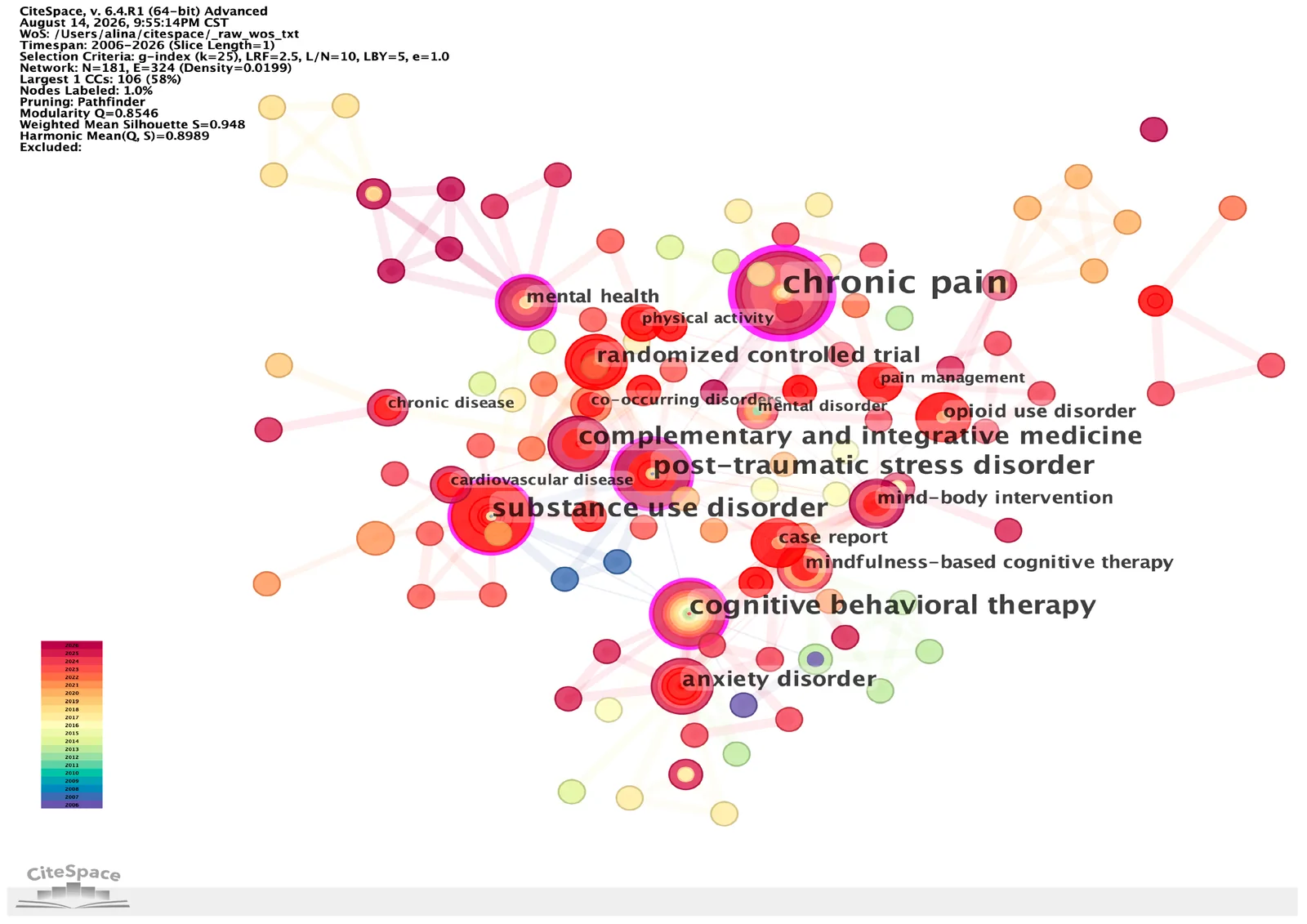
Keyword co-occurrence network in research on mind–body therapies for multimorbidity. Nodes represent author keywords, and larger nodes indicate more frequent keywords. Links represent keyword co-occurrence within the same publication, with thicker links indicating stronger relationships. Colours correspond to publication years.

**Figure 5:**
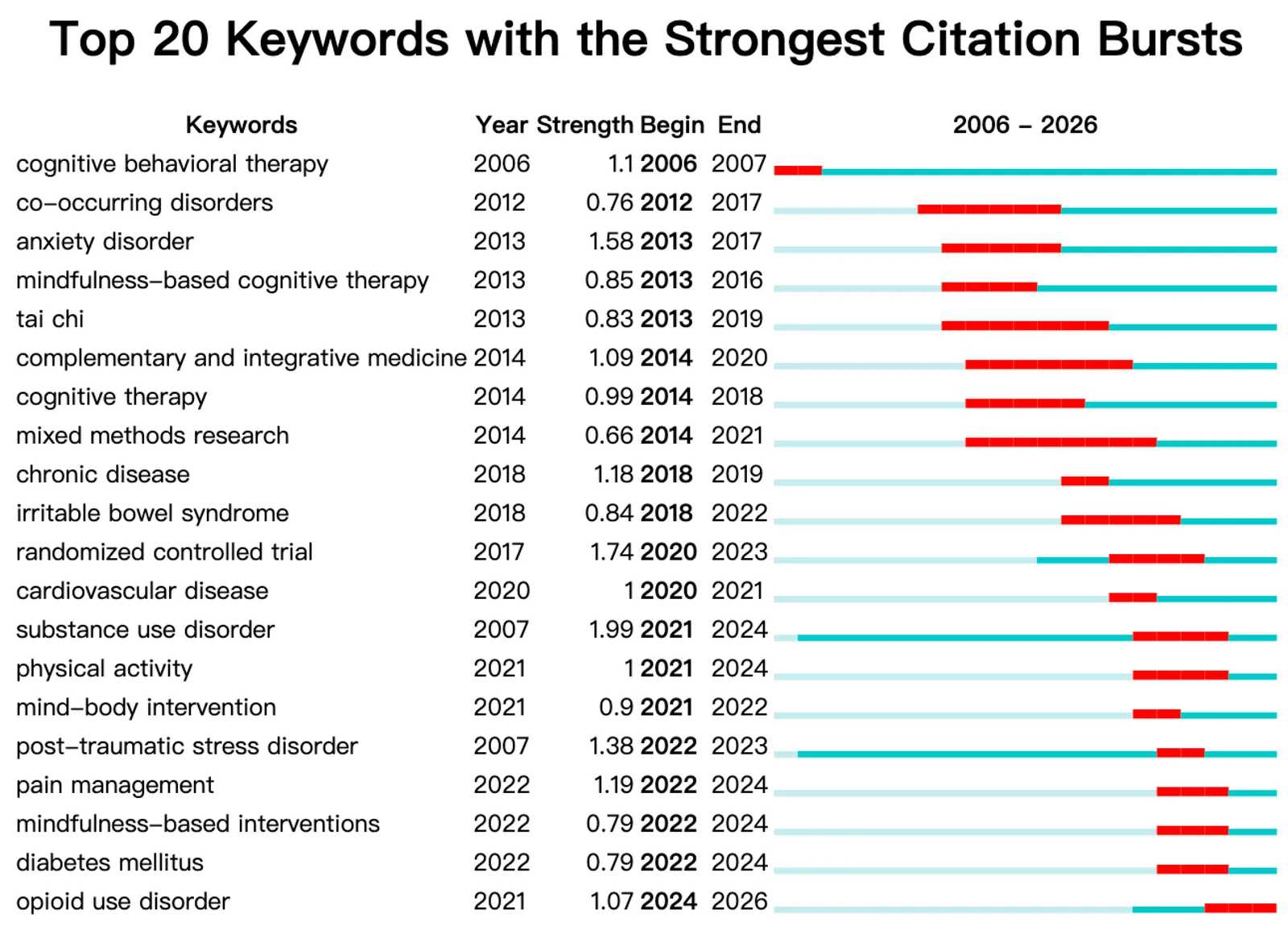
Top 20 keywords with the strongest citation bursts in research on mind–body therapies for multimorbidity. Year indicates the first occurrence of each keyword, and Strength represents burst intensity. The light blue segments indicate the years before a keyword’s first occurrence; the darker blue segments indicate the periods after its first occurrence but outside the detected burst interval; and the red segments indicate the detected burst period between Begin and End. Data for 2026 cover January through July only.

Keyword co-occurrence analysis characterised the main research topics and their development over time (Figure 4 and Figure 5). The network contained 181 nodes and 324 links, with a density of 0.0199. The most frequent keywords were “chronic pain” (*n* = 18), “cognitive behavioural therapy” (*n* = 10), “substance use disorder” (*n* = 10), “post-traumatic stress disorder” (n = 9), “complementary and integrative medicine” (*n* = 8), “randomised controlled trial” (*n* = 6), and “anxiety disorder” (*n* = 6). The highest betweenness centrality values were observed for “chronic pain” (0.26), “substance use disorder” (0.18), and “cognitive behavioural therapy” (0.14), indicating bridging positions in the keyword network rather than clinical importance or treatment efficacy. Burst analysis indicated that earlier research focused on cognitive and mindfulness-based therapies, anxiety, and co-occurring disorders. More recent bursts involved randomised controlled trials, substance use disorder, physical activity, pain management, and mindfulness-based interventions. The strongest recent bursts were “substance use disorder” (strength = 1.99, 2021 to 2024) and “randomised controlled trial” (strength = 1.74, 2020 to 2023). “Opioid use disorder” had an ongoing burst from 2024 through 2026, although the partial coverage of 2026 warrants caution. These patterns describe changes in research attention, not comparative treatment efficacy. “Cognitive behavioural therapy” also occurred in publications on mindfulness-based cognitive therapy and as a comparator, co-intervention, or background concept. Conventional cognitive behavioural therapy alone was not classified as an eligible mind–body therapy.

**Figure 6:**
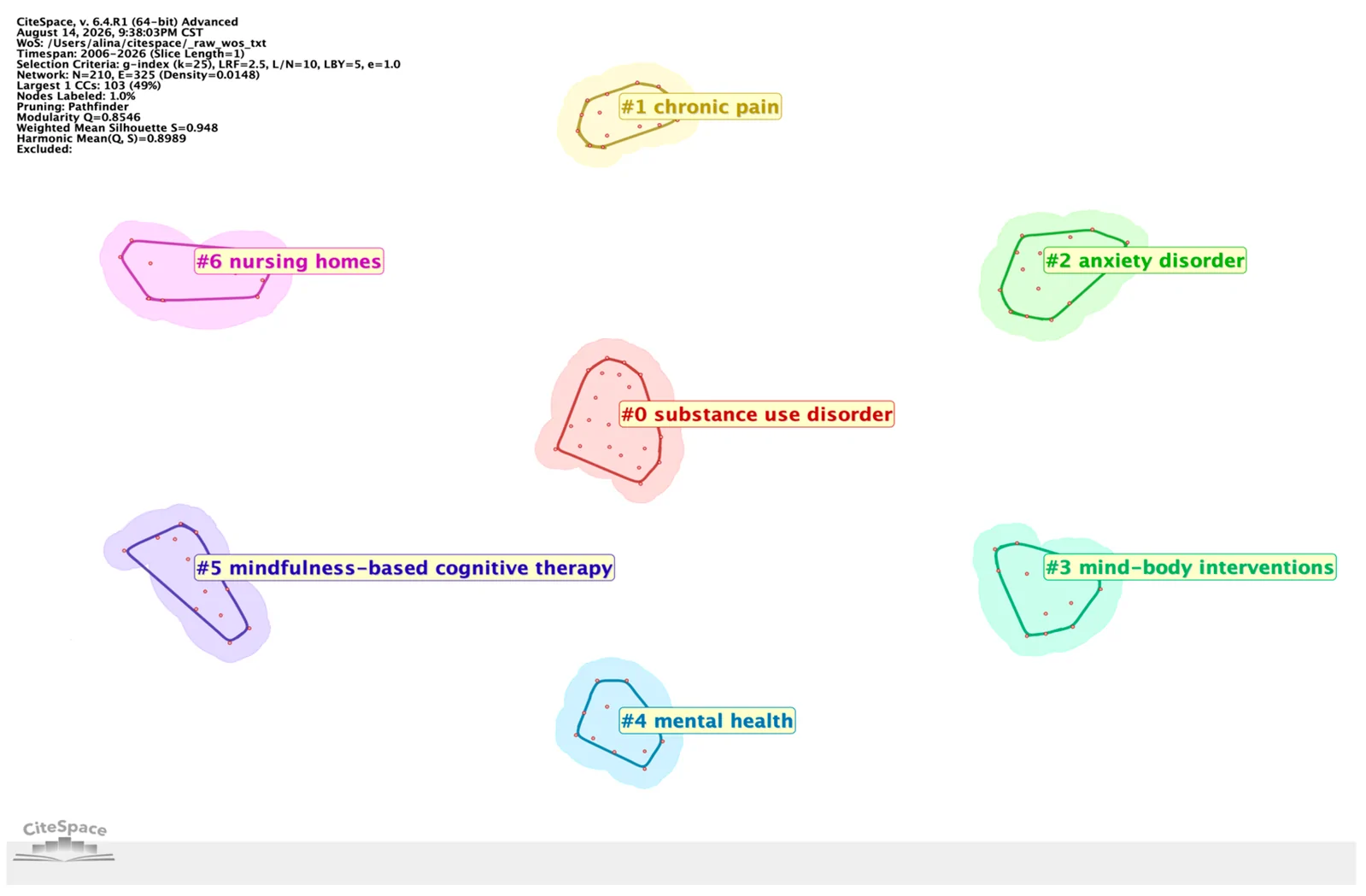
Keyword cluster network in research on mind–body therapies for multimorbidity. Nodes represent keywords, and links indicate keyword co-occurrence. Coloured areas represent clusters generated using the log-likelihood ratio method. Clusters are numbered in descending order of size, beginning with #0.

The clustering analysis used a network of 210 nodes and 325 links, with a density of 0.0148 (Figure 6). The modularity Q value was 0.8546 and the weighted mean silhouette value was 0.948, consistent with clear separation among clusters and high internal consistency. The following seven clusters were identified: “substance use disorder” (#0), “chronic pain” (#1), “anxiety disorder” (#2), “mind–body interventions” (#3), “mental health” (#4), “mindfulness-based cognitive therapy” (#5), and “nursing homes” (#6). Together, the clusters covered substance-related and anxiety disorders, chronic pain, specific mind–body approaches, broader mental health topics, and institutional care. The labels describe the thematic structure of the publications and do not establish the clinical importance of any intervention or research area.

**Figure 7:**
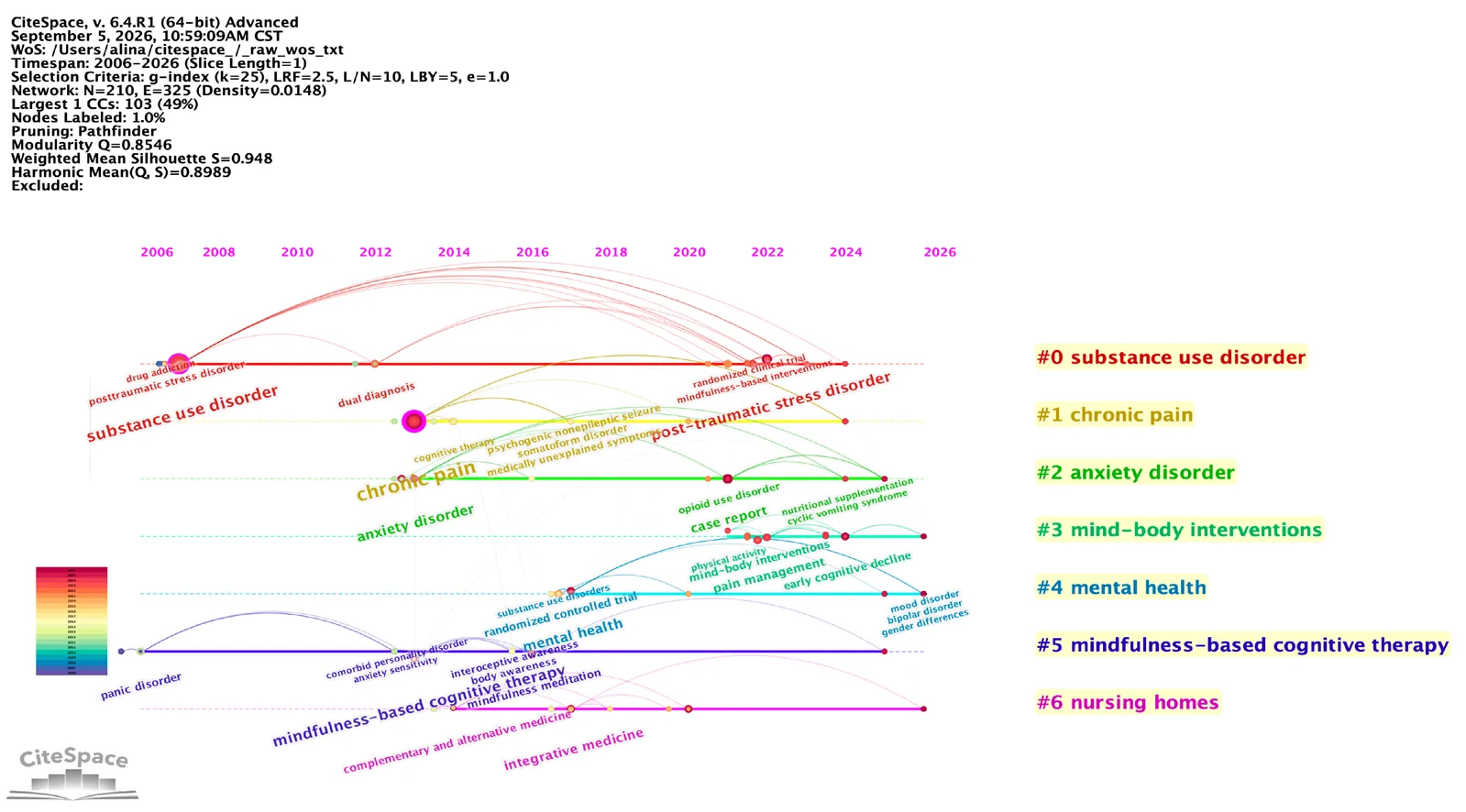
Timeline visualisation of keyword clusters in research on mind–body therapies for multimorbidity. Each horizontal line represents one keyword cluster, and the horizontal axis indicates publication year. Keywords are positioned according to their temporal occurrence, while links show relationships among keywords. Node and link colours correspond to publication years, whereas the coloured horizontal lines and cluster labels distinguish clusters #0–#6.

The timeline visualisation shows how these thematic clusters developed during the study period (Figure 7). Early activity was concentrated in the “substance use disorder” and “mindfulness-based cognitive therapy” clusters, with topics that included addiction, post-traumatic stress disorder, panic disorder, and behavioural treatment. The “chronic pain” and “anxiety disorder” clusters became more prominent in the middle of the period. Later publications extended to broader mind–body interventions, pain management, opioid use disorder, mental health, and institutional or community-based applications. The timeline shows changes in the distribution of research topics across clinical conditions, settings, and designs. It does not demonstrate a conceptual shift in the management of multiple coexisting diagnosed conditions or the comparative effectiveness of individual mind–body therapies.

### 3.4. Distribution of Authors and Institutions

**Figure 8:**
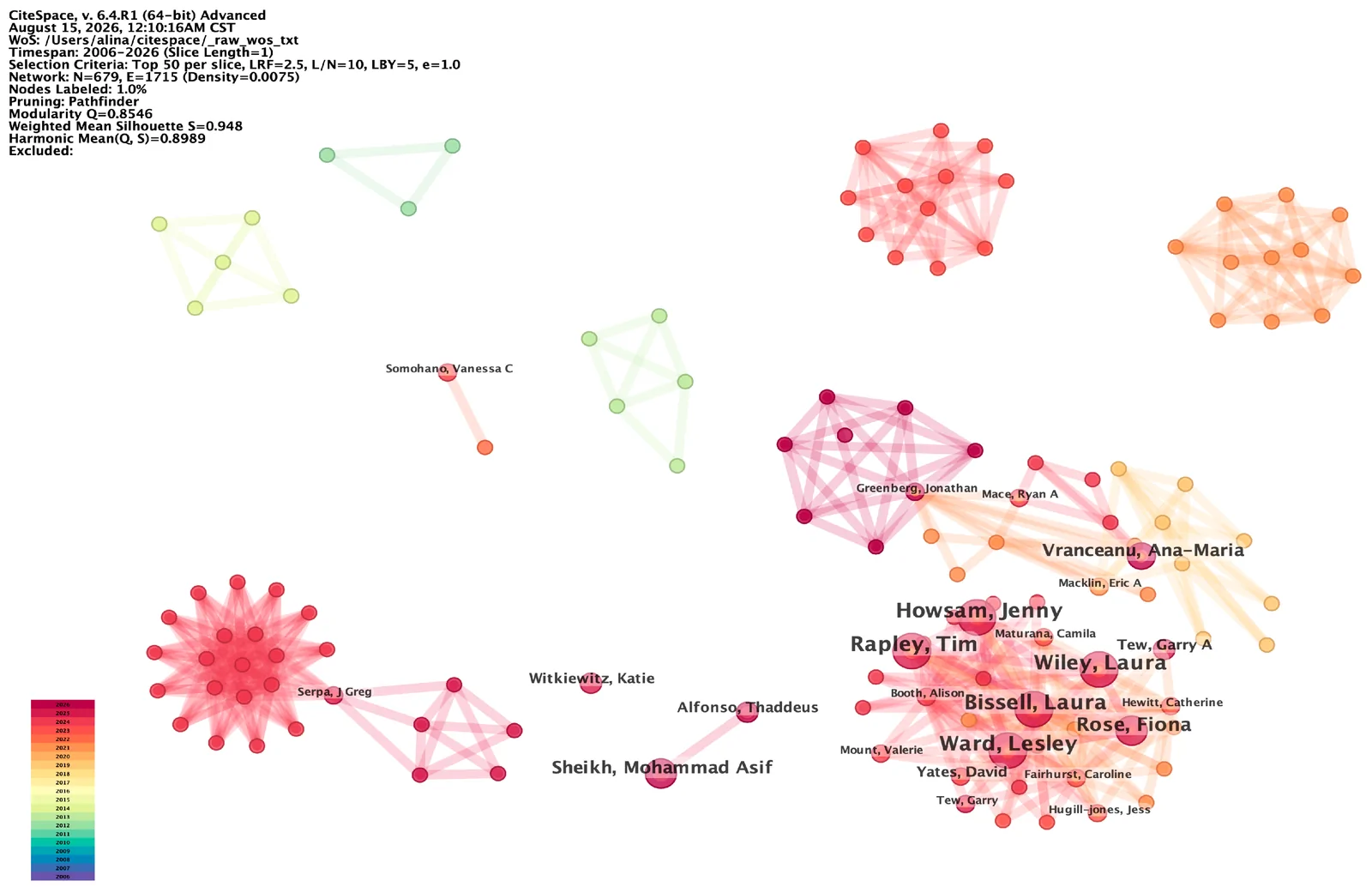
Author collaboration network in research on mind–body therapies for multimorbidity. Nodes represent authors, and node size indicates the number of included publications. Links represent coauthorship, with thicker links indicating stronger collaboration. Colours correspond to publication years.

The author collaboration network included 679 authors and 1715 links, with a density of 0.0075 (Figure 8). Table 1 lists the eight most frequently represented authors and their standardised affiliations. Lesley Ward, Laura Bissell, Laura Wiley, Jenny Howsam, and Tim Rapley each contributed six publications; Fiona Rose contributed five; and Ana-Maria Vranceanu and Mohammad Asif Sheikh contributed four each. Six of these eight authors were affiliated with institutions in the United Kingdom, particularly Northumbria University, the University of York, and British Wheel of Yoga Qualifications. One large group included Ward, Bissell, Wiley, Howsam, Rapley, Rose, and Garry A. Tew. Other authors formed several smaller or disconnected groups. The low density was consistent with collaboration occurring mainly within individual research teams, with relatively few links between teams. These measures describe collaboration within this dataset and should not be interpreted as indicators of author influence, research quality, or scientific leadership.

**Figure 9:**
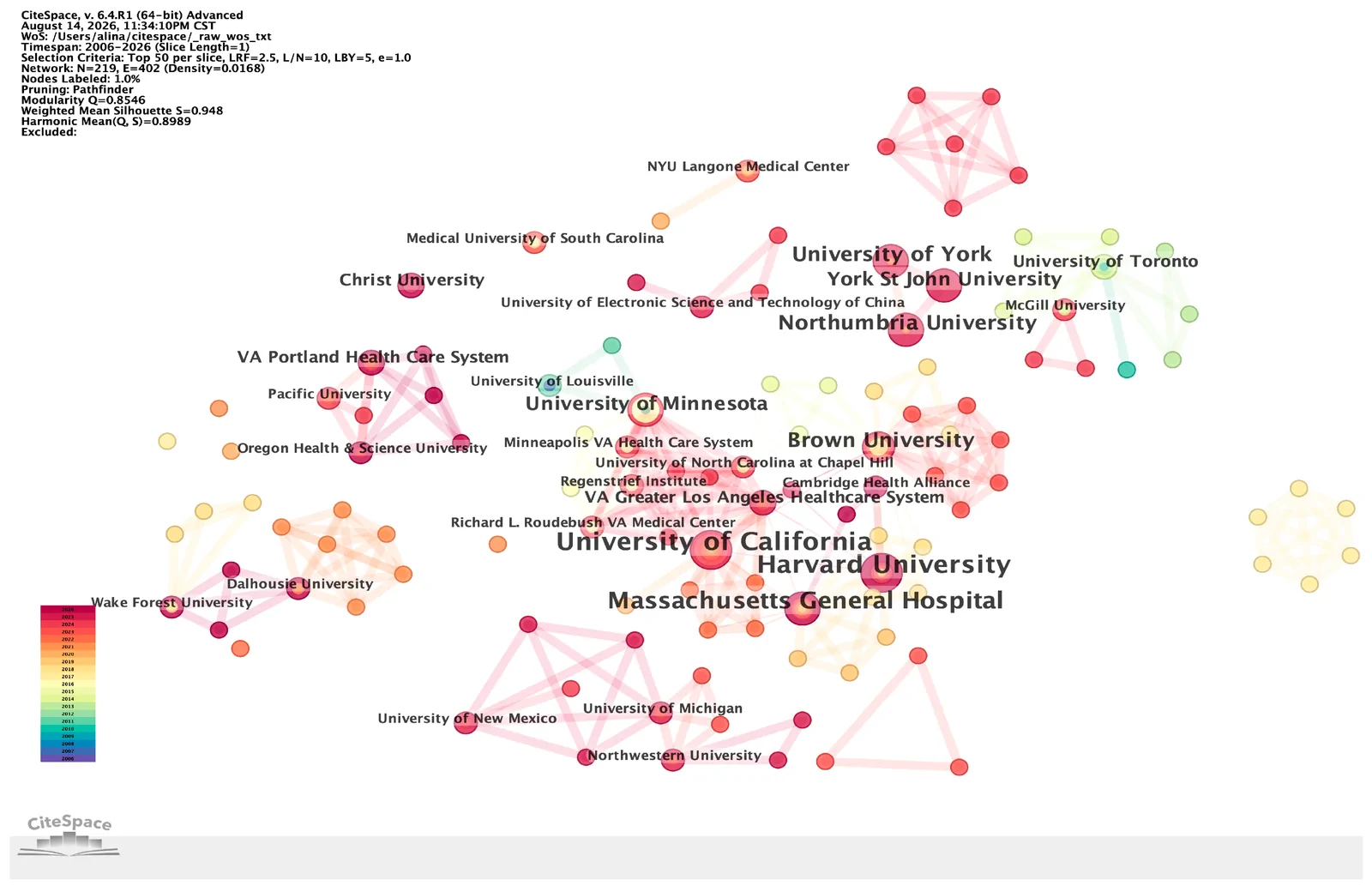
Institutional collaboration networks in research on mind–body therapies for multimorbidity. Nodes represent standardised institutional affiliations, and node size indicates the number of included publications. Links represent interinstitutional collaboration, with thicker links indicating stronger collaboration. Colours correspond to publication years.

The institutional collaboration network contained 219 nodes and 402 links, with a density of 0.0168 (Figure 9). Table 2 lists the eight most frequently represented institutions. The University of California and Harvard University each contributed seven publications, followed by Massachusetts General Hospital with six. Brown University, the University of York, and Northumbria University each contributed five publications; the University of Minnesota and York St John University each contributed four. A large North American group included Harvard University, Massachusetts General Hospital, the University of California, Brown University, and several Veterans Affairs healthcare organisations. Another group included the University of York, Northumbria University, and York St John University. Several smaller components were disconnected from the larger groups. The low density was consistent with limited cross-institutional collaboration within the included publications. Among the eight most frequently represented institutions, betweenness centrality ranged from 0.00 to 0.03. Brown University had the highest value (0.03), followed by the University of California (0.02). Harvard University, Massachusetts General Hospital, and the University of Minnesota each had a value of 0.01. These values represent bridging positions in the present network, not institutional influence, scientific leadership, or research quality.

### 3.5. Journal Distribution and Co-Citation Analysis

**Figure 10:**
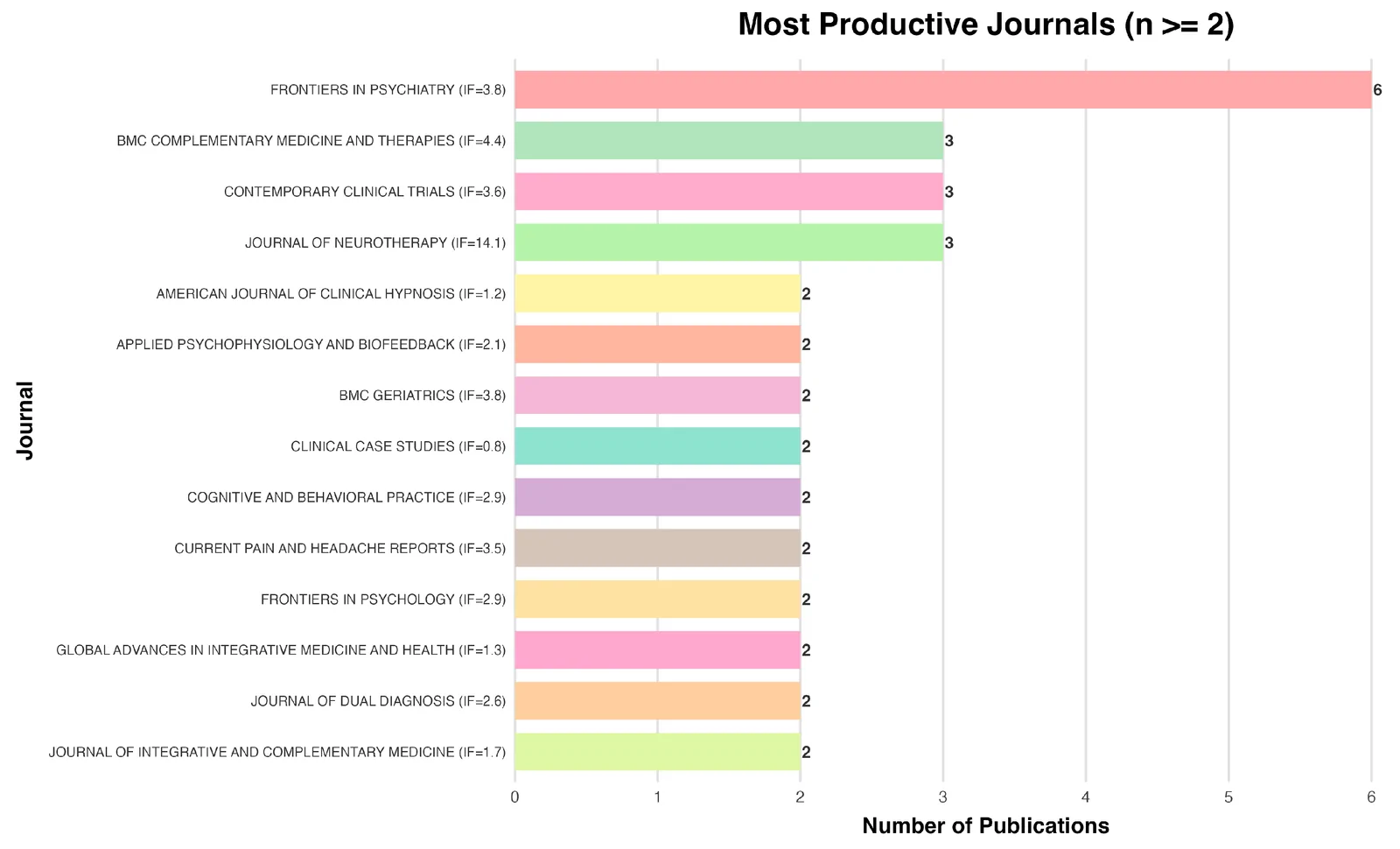
Journal distribution in research on mind–body therapies for multimorbidity. Bars represent journals with at least two included publications, and bar length indicates publication count. IF = journal impact factor for 2025.

The 119 publications appeared in 98 standardised journal titles, indicating wide dispersion across publication sources. Fourteen journals published at least two of the included publications and are shown in Figure 10. *Frontiers in Psychiatry* published the largest number, with six (6/119, 5.04%). *BMC Complementary Medicine and Therapies*, *Contemporary Clinical Trials*, and *Journal of Neurotherapy* each published three (3/119, 2.52%); the other ten journals in Figure 10 each published two (2/119, 1.68%). The journals covered psychiatry, psychology, pain, complementary and integrative medicine, geriatrics, clinical trial methods, hypnosis, and biofeedback.

**Figure 11:**
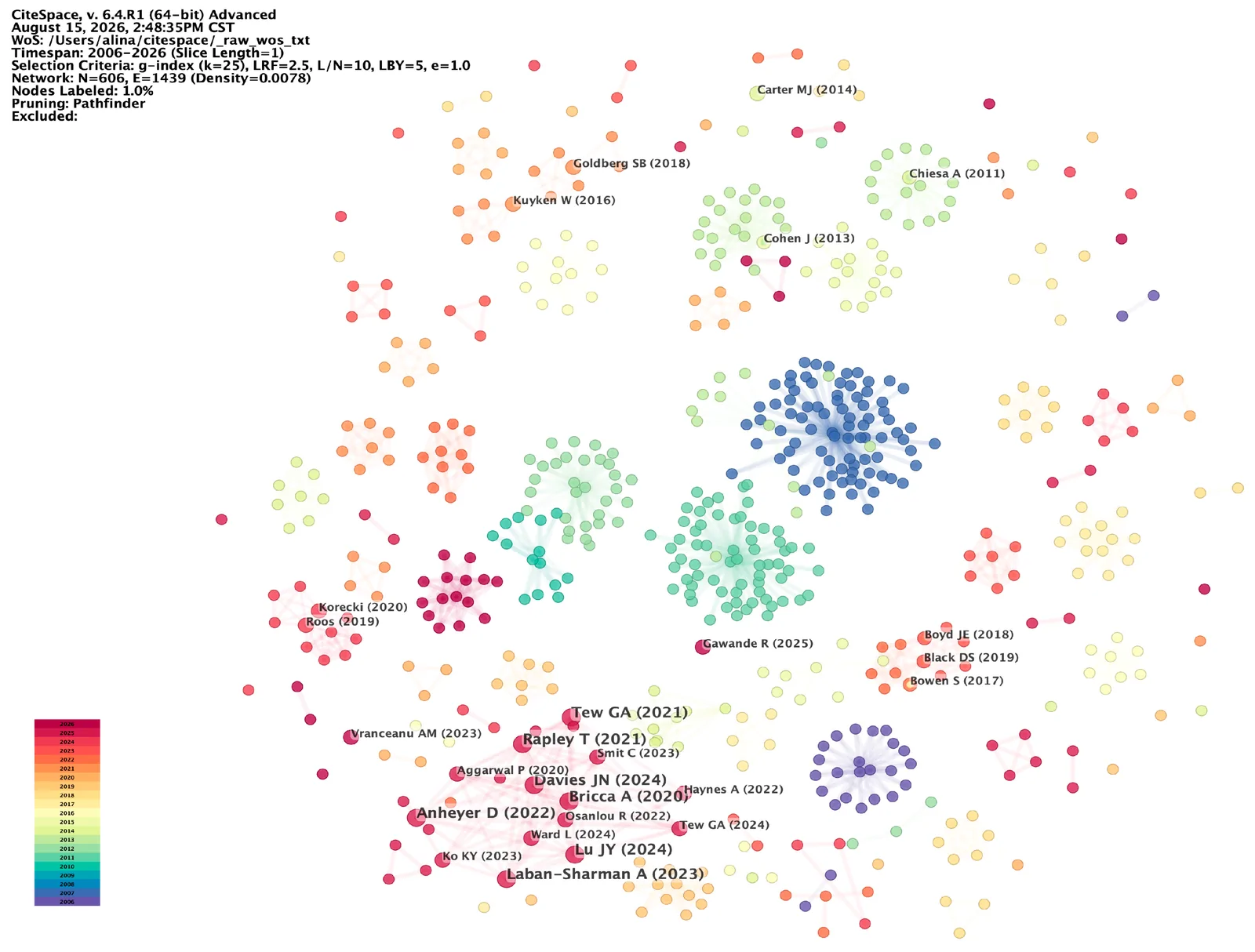
Co-cited reference network in research on mind–body therapies for multimorbidity. Nodes represent cited references, and node size indicates citation frequency within the included dataset. Links connect references cited together in the same publication, with thicker links indicating stronger co-citation relationships. Colours indicate citation time slices, and node labels use the Author + Year format to identify cited references.

The co-cited reference network (Figure 11) contained 606 nodes and 1439 links, with a density of 0.0078. Each node represents a cited reference; node size indicates citation frequency within the dataset, and link thickness indicates co-citation strength. Co-citation identifies sources cited together and does not indicate that a source provides clinical trial evidence. Seven references shared the highest co-citation frequency, with three citations each (Table 3). The following four were evidence syntheses: a systematic review of yoga for anxiety in adults, separate meta-analyses of yoga for knee osteoarthritis and low back pain, and a systematic review and meta-analysis of exercise therapy for people with multimorbidity. The other three were a population-based analysis of 20-year trends in meditation, yoga, guided imagery, and progressive relaxation among US adults; the protocol for the Gentle Years Yoga randomised controlled trial in older adults with multimorbidity; and a book chapter on qualitative data analysis. The most frequently co-cited literature therefore concerned yoga and exercise-based interventions across mental and physical conditions, complementary health approaches, and qualitative methods.

### 3.6. Clinical Evidence Mapping

**Figure 12:**
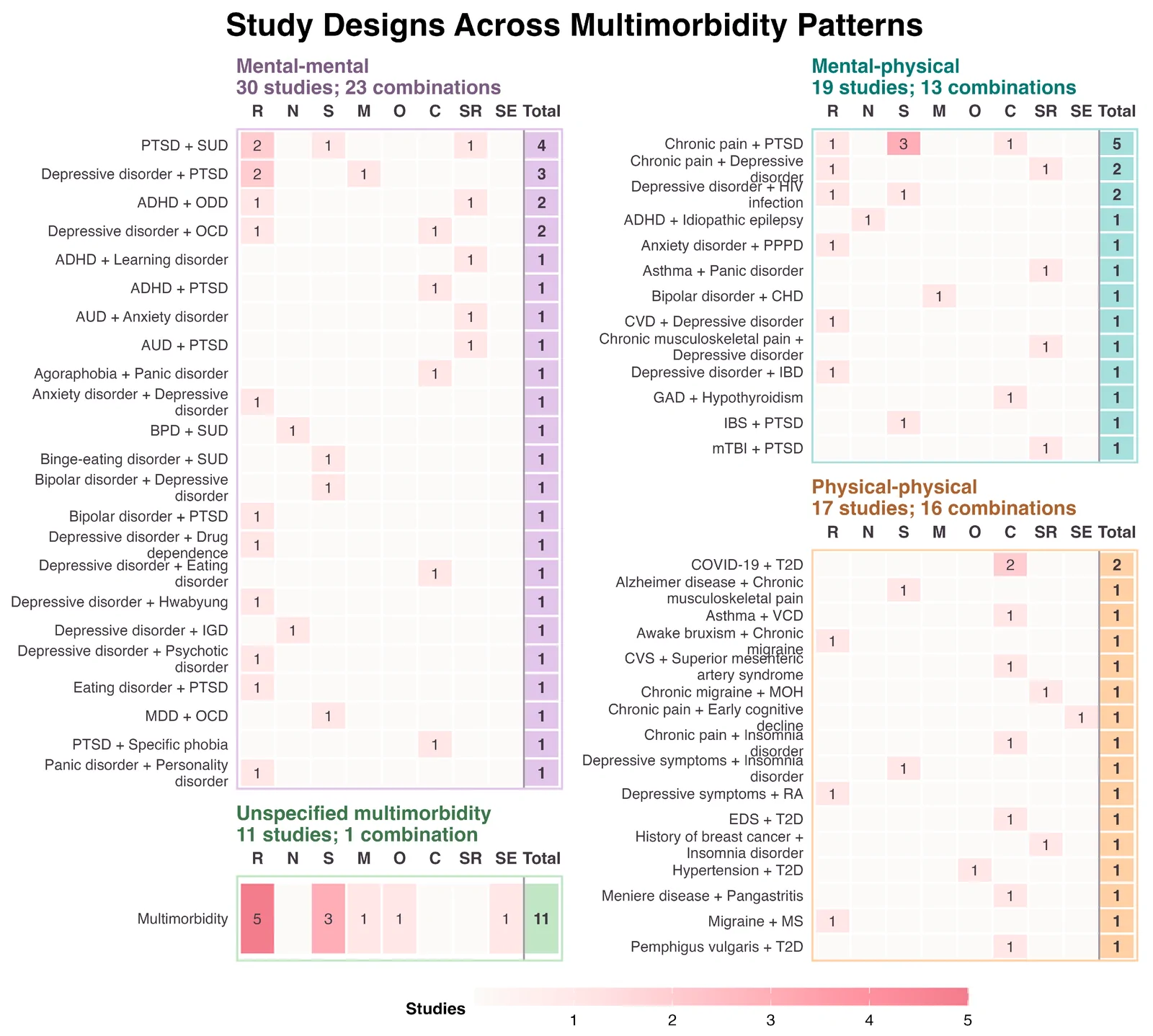
Multimorbidity combination patterns and study designs. Rows represent specific condition combinations, columns represent study designs, and cell values and colour intensity indicate the number of clinical studies. The Total column gives the total for each condition combination. R = randomised controlled trial; N = non-randomised controlled trial; S = single-arm intervention study; M = mixed-method intervention study; O = observational study; C = case report or case series; SR = secondary analysis of a randomised trial; SE = secondary or exploratory analysis. ADHD = attention-deficit/hyperactivity disorder; AUD = alcohol use disorder; BPD = borderline personality disorder; CHD = coronary heart disease; COVID-19 = coronavirus disease 2019; CVD = cardiovascular disease; CVS = cyclic vomiting syndrome; EDS = Ehlers–Danlos syndrome; GAD = generalised anxiety disorder; HIV = human immunodeficiency virus; IBD = inflammatory bowel disease; IBS = irritable bowel syndrome; IGD = internet gaming disorder; MDD = major depressive disorder; mTBI = mild traumatic brain injury; MOH = medication-overuse headache; MS = multiple sclerosis; OCD = obsessive–compulsive disorder; ODD = oppositional defiant disorder; PPPD = persistent postural-perceptual dizziness; PTSD = post-traumatic stress disorder; RA = rheumatoid arthritis; SUD = substance use disorder; T2D = type 2 diabetes; VCD = vocal cord dysfunction.

Across the 77 clinical studies, 53 specific combinations of coexisting diagnosed conditions were identified (Figure 12). Mental–mental combinations were most common (30/77, 39.0%), followed by mental–physical (19/77, 24.7%) and physical–physical combinations (17/77, 22.1%). Eleven clinical studies reported multimorbidity without sufficient information to identify a specific condition pair (11/77, 14.3%). Among identifiable pairs, chronic pain with post-traumatic stress disorder (PTSD) was the most frequently studied (5/77, 6.5%), followed by PTSD with substance use disorder (4/77, 5.2%) and depressive disorder with PTSD (3/77, 3.9%). Five other combinations were examined in two clinical studies each, whereas most specific combinations appeared in only one.

Randomised controlled trials were the most common design (27/77, 35.1%). Case reports or case series accounted for 15 clinical studies (15/77, 19.5%), single-arm intervention studies for 14 (14/77, 18.2%), and secondary analyses of randomised trials for 11 (11/77, 14.3%). Non-randomised controlled trials and mixed-method intervention studies each contributed three clinical studies (3/77, 3.9%), while observational studies and secondary or exploratory analyses each contributed two (2/77, 2.6%). Secondary analyses of randomised trials used data from completed randomised trials; the secondary or exploratory category comprised intervention-related analyses that did not meet this criterion. The two observational studies included delivery of an eligible intervention and therefore met the clinical evidence-map criteria. Figure 12 shows the designs represented within each condition combination, with detailed counts in Supplementary Table S5.

**Figure 13:**
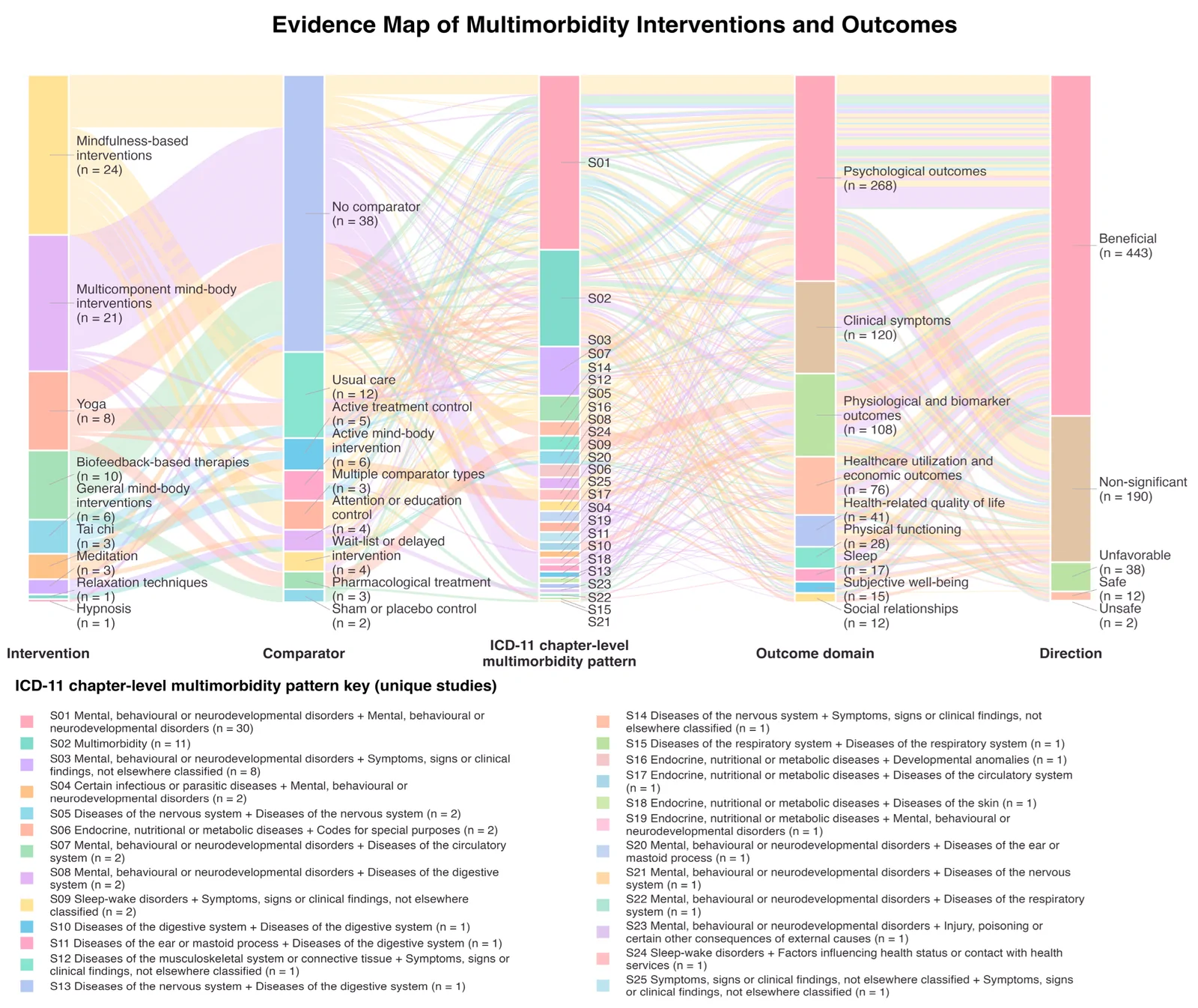
Evidence-map Sankey diagram of interventions, multimorbidity patterns, and outcomes. Counts in the intervention, comparator, and multimorbidity-pattern columns represent unique clinical studies (N = 77). Counts in the outcome-domain and outcome-direction columns, as well as ribbon widths, represent extracted outcome occurrences (n = 685), because one study could contribute multiple outcomes. Ribbon colours indicate intervention categories. ICD-11 = International Classification of Diseases, Eleventh Revision.

The Sankey diagram links intervention types and comparator categories to ICD-11 disease-system combinations, outcome domains, and outcome directions across the 77 clinical studies (Figure 13). Mindfulness-based interventions were most common (24/77, 31.2%), followed by multicomponent mind–body interventions (21/77, 27.3%), biofeedback-based therapies (10/77, 13.0%), yoga (8/77, 10.4%), general mind–body interventions (6/77, 7.8%), meditation (3/77, 3.9%), tai chi (3/77, 3.9%), hypnosis (1/77, 1.3%), and relaxation techniques (1/77, 1.3%). Thirty-eight clinical studies had no comparator (38/77, 49.4%). Comparator categories were usual care (12/77, 15.6%), an active mind–body intervention (6/77, 7.8%), an active treatment control (5/77, 6.5%), an attention or education control (4/77, 5.2%), a wait-list or delayed intervention (4/77, 5.2%), multiple comparator types (3/77, 3.9%), pharmacological treatment (3/77, 3.9%), and a sham or placebo control (2/77, 2.6%).

The 53 specific condition combinations corresponded to 25 nondirectional ICD-11 disease-system combinations. Combinations involving two mental, behavioural, or neurodevelopmental conditions were most common (30/77, 39.0%), followed by unspecified multimorbidity (11/77, 14.3%) and combinations of mental, behavioural, or neurodevelopmental disorders with symptoms, signs, or clinical findings not elsewhere classified (8/77, 10.4%). Each remaining ICD-11 system combination appeared in one or two clinical studies. Complete intervention, comparator, and disease-system distributions are reported in Supplementary Table S3.

Psychological outcomes were the most frequently assessed domain (65/77, 84.4%), followed by clinical symptoms (48/77, 62.3%), healthcare utilisation or economic outcomes (39/77, 50.6%), physiological or biomarker outcomes (27/77, 35.1%), physical functioning (16/77, 20.8%), health-related quality of life (15/77, 19.5%), subjective well-being (13/77, 16.9%), sleep (11/77, 14.3%), and social relationships (8/77, 10.4%). All percentages use the 77 clinical studies as the denominator. Because a clinical study could assess more than one domain, the categories were not mutually exclusive and the percentages do not sum to 100%. Clinical studies assessed a median of three outcome domains (interquartile range, 2 to 4; range, 1 to 7), and 67 assessed at least two domains (67/77, 87.0%).

At least one beneficial finding was reported in 75 clinical studies (75/77, 97.4%), while 51 reported at least one non-significant finding (51/77, 66.2%) and 28 reported at least one unfavourable finding (28/77, 36.4%). Beneficial findings most often involved psychological outcomes, whereas unfavourable findings most often involved healthcare utilisation or economic outcomes. Twelve clinical studies reported favourable safety findings (12/77, 15.6%), and two reported safety concerns involving clinical symptoms (2/77, 2.6%). Detailed outcome-domain and direction distributions are provided in Supplementary Table S6. Because outcome domains and directions were not mutually exclusive, these findings should not be interpreted as pooled estimates of efficacy or safety.

## 4. Discussion

This study combined bibliometric analysis with evidence mapping to examine publications on mind–body therapies for people with comorbidity and multimorbidity. The bibliometric corpus comprised 119 publications, of which 77 clinical studies were included in the evidence map. Publication output was higher after 2017, but the literature remained dispersed across countries, journals, research teams, and condition combinations. Research topics centred mainly on chronic pain, post-traumatic stress disorder (PTSD), substance use disorders, and anxiety disorders, while the clinical studies most often examined mental–mental and mental–physical combinations. The relevance of mind–body therapies to multimorbidity lies in their potential to address symptoms and functional problems that cross disease boundaries, not in established evidence of comparative effectiveness. Multimorbidity involves interactions between conditions, substantial treatment burden, and multidimensional outcomes that may not be adequately addressed by interventions directed at a single disease [25,26].

The literature was geographically concentrated as follows: publications with a US first author accounted for 52.94%, and only 15.13% involved international collaboration. The author and institutional collaboration networks both had low densities, with most links occurring within relatively independent research teams. The 119 publications appeared in 98 journals spanning psychiatry, pain medicine, geriatrics, psychology, complementary and integrative medicine, and clinical trial methodology. This distribution places relevant work across several clinical and methodological communities rather than within one specialist literature, which may contribute to variation in terminology, classification methods, and outcome measures. National publication counts, network centrality, and collaboration links describe the structure of this dataset; they do not measure research quality, academic leadership, or clinical influence.

The keyword and clustering results were broadly consistent with the condition composition of the evidence map. Chronic pain, substance use disorder, and PTSD were prominent in the keyword network, and the most common specific combinations included chronic pain with PTSD, PTSD with substance use disorder, and depressive disorder with PTSD. Recent bursts involving randomised controlled trials, pain management, mindfulness-based interventions, substance use disorder, and opioid use disorder indicate changes in research attention, not changes in clinical practice or comparative effectiveness. The frequency of cognitive behavioural therapy also requires interpretation in light of the inclusion criteria. Several publications concerned mindfulness-based cognitive therapy, which combines mindfulness practice with elements of cognitive therapy [27]. Cognitive behavioural therapy also appeared as a comparator, co-intervention, or background concept. Conventional cognitive behavioural therapy alone was not an eligible mind–body intervention. Its frequency in the keyword network therefore does not indicate that conventional cognitive behavioural therapy publications were included or permit a comparison with mind–body therapies.

The 77 clinical studies covered 53 specific condition combinations, most of which appeared in only one clinical study. Mental–mental combinations accounted for 39.0%, mental–physical combinations for 24.7%, and physical–physical combinations for 22.1%. Research was concentrated at the intersections of pain, trauma, mood disorders, and addiction, while combinations involving cardiovascular, metabolic, respiratory, musculoskeletal, and other physical conditions were less well-represented. This distribution does not show that frequently studied combinations are more suitable for mind–body therapies or have greater clinical priority. Rather, the 53 combinations across 77 clinical studies show that most clinical contexts lack independent replication, limiting generalisation from one condition combination to another.

Mindfulness-based interventions were the most frequently studied approach, accounting for 31.2%, followed by multicomponent mind–body interventions, biofeedback-based therapies, and yoga. This distribution cannot determine which mind–body therapy is more effective. Twenty-seven clinical studies used a randomised controlled design, but 38 had no comparator, and case reports, case series, and single-arm studies were common. Even among controlled studies, usual care, active mind–body interventions, and other active treatments address different comparison questions. The clinical studies also differed in condition combinations, intervention dose, comparator selection, follow-up, and outcome measurement. Consequently, Sankey flows, the number of clinical studies in an intervention category, and the frequency of favourable findings cannot provide estimates of comparative effectiveness. Direct comparative trials or meta-analyses of sufficiently comparable clinical studies are required to determine which therapy works better for a particular population.

Biofeedback-based therapies accounted for 13.0% of the evidence map, and neurofeedback warrants separate consideration within the broader mind–body framework. Neurofeedback uses real-time information about neural activity to support learned self-regulation and is therefore related conceptually to mind–body therapies [28]. Its equipment and feedback procedures nevertheless differ from the attention, breathing, and movement practices used in mindfulness, yoga, and tai chi. These approaches should not be treated as interchangeable. Future neurofeedback studies may use the CRED-nf checklist to improve consistency in study design and reporting [29].

The included clinical studies recognised some of the multidimensional nature of multimorbidity. Psychological outcomes and clinical symptoms were assessed in 84.4% and 62.3% of clinical studies, respectively; healthcare utilisation or economic outcomes were assessed in 50.6% and physiological or biomarker outcomes in 35.1%. The median number of outcome domains was three, and 87.0% of clinical studies included at least two. This breadth is more consistent with the clinical complexity of multimorbidity than an exclusive focus on symptoms associated with one disease. Some clinical studies also extended assessment beyond patient-reported psychological outcomes to healthcare utilisation, economic measures, and biomarkers. However, multiple domains do not ensure that the selected outcomes were comparable or clinically important. Because several domains could be recorded within one clinical study, these percentages are not mutually exclusive and should not be added as a single composition.

Outcome directions also require careful interpretation. Although 75 clinical studies reported at least one beneficial finding, 51 reported at least one non-significant finding and 28 reported at least one unfavourable finding. These categories could overlap within the same clinical study, particularly when several outcomes or time points were reported. The finding that 97.4% of clinical studies reported at least one beneficial result is therefore neither an overall efficacy rate nor a pooled effect estimate, and it provides no information about effect size, precision, or clinical importance. Unfavourable findings were most often recorded for healthcare utilisation or economic outcomes, but this pattern cannot show that improvements in symptoms or psychological well-being failed to reduce healthcare use or costs because the findings may derive from different clinical studies or outcomes. Safety outcomes were reported in relatively few clinical studies, and absence of reported adverse events should not be interpreted as evidence of safety. Publication bias and selective outcome reporting may also increase the visibility of favourable findings [30].

The proposed mechanisms of mind–body therapies in people with multimorbidity are derived mainly from basic research, clinical trials, and evidence syntheses outside the present study; they were not tested by this bibliometric analysis. Mindfulness and related interventions may influence responses to stress, pain, and negative emotions through attention regulation, decentring, emotion regulation, and body awareness [31,32,33]. Yoga and mindfulness-based stress reduction may also affect autonomic activity and stress-related physiological measures through breathing, movement, and relaxation. These processes may be relevant because pain, anxiety, sleep disturbance, and functional limitations often cross diagnostic categories. However, the external literature differs considerably in control conditions, sample sizes, intervention doses, and biomarker selection [34]. These pathways provide a rationale for further study, not evidence that one intervention can modify several diseases simultaneously.

Chronic low-grade inflammation is associated with cardiovascular, metabolic, neuropsychiatric, and age-related conditions and may contribute to the co-occurrence of several diseases [35]. Some reviews have reported associations between mindfulness training and changes in inflammatory or immune markers, but the evidence remains preliminary, with modest and inconsistent effects [36,37]. Differences in participants, intervention content, follow-up, and laboratory measures make the literature difficult to interpret. The cross-disease role of inflammation does not establish that mind–body therapies can treat several conditions simultaneously. Stress regulation, autonomic function, emotion regulation, and inflammation are better treated as candidate pathways for direct investigation in people with multimorbidity. Evidence of mediation would be more informative than a change in a biomarker measured before and after treatment.

These proposed mechanisms require testing in well-designed clinical studies. Future studies should prespecify mechanistic measures and examine whether changes mediate clinical outcomes, rather than measuring several biomarkers before and after treatment without a mediation analysis. Because mind–body therapies often contain interacting components, their effects may depend on intervention dose, practitioner qualifications, participant engagement, cultural context, and healthcare setting. The complex intervention framework may help researchers evaluate these factors alongside clinical outcomes [38]. Clinical studies involving people with multimorbidity should also consider condition combinations, baseline functioning, treatment burden, and individual treatment goals. Outcome selection may draw on the Core Outcome Set for Multimorbidity, including quality of life, mental health, physical function, treatment burden, healthcare utilisation, and adverse events [39]. Interventions with a physical activity component, such as yoga and tai chi, should report both benefits and harms [40].

This study differs from previous bibliometric analyses in its target population and unit of analysis. Previous studies examined mind–body therapies broadly or focused on individual therapies such as tai chi [10,41]. The present study was restricted to people with two or more formally diagnosed conditions and added study-level coding of condition combinations, intervention types, comparators, outcome domains, and outcome directions. Combining bibliometric analysis with evidence mapping allowed the thematic structure of the field to be considered alongside the design and clinical scope of the underlying clinical studies. The Sankey diagram and condition map identify which combinations have been studied, which designs have been used, and where evidence is sparse; they do not rank interventions. This study-level clinical evidence map is the main addition to previous bibliometric analyses of mind–body therapies.

Future research should prioritise adequately powered multicentre randomised trials in clinically important condition combinations. Usual care, attention controls, or other active treatments should be used as comparators, with sufficient follow-up to determine whether effects are sustained. Direct comparisons between mind–body therapies and component analyses of multicomponent interventions may help identify which elements contribute to observed outcomes. Trials should distinguish effects on individual conditions, shared symptoms, physical functioning, and overall treatment burden. Research should include a wider range of physical condition combinations, age groups, and low- and middle-income settings; standardise intervention classifications and outcome definitions; and report adherence, co-interventions, adverse events, and healthcare utilisation. Broader international collaboration would improve geographical diversity and allow findings to be examined across health systems and cultural contexts.

This study has several strengths. First, the search covered WoSCC, PubMed, and Embase, three complementary databases, and required at least two formally diagnosed conditions. Publications in which a single disease was accompanied only by general mood, stress, or sleep symptoms were excluded, improving consistency with the definitions of comorbidity and multimorbidity. Second, the study combined knowledge-structure analyses of 119 publications with an evidence map of 77 clinical studies, avoiding clinical inferences based only on keywords or citation networks. Condition combinations, interventions, comparators, and outcomes were coded using a predefined framework, and the main categories were reported with absolute numbers and percentages. Reporting the number of outcome domains per clinical study also made their non-mutually exclusive structure explicit. The study distinguished research frequency, network position, outcome direction, and comparative effectiveness, reducing the risk of interpreting bibliometric prominence as clinical evidence. Independent screening, agreement assessment, partial duplicate data extraction, and availability of study-level data improved transparency and reproducibility.

Several limitations should be considered. The search began in 2004, so earlier publications and the initial development of the field were not captured. Only English-language publications were included, and differences in database coverage may have led to the omission of publications from regional journals or non-English-speaking settings. These restrictions may also have contributed to the observed geographical concentration. Although author, institution, and keyword names were standardised, name variants, institutional changes, and differences in terminology may still have affected the networks. Bibliometric indicators are also influenced by publication date and the time available for citations to accumulate; they cannot assess methodological quality, risk of bias, or clinical importance.

The evidence map did not assess risk of bias, grade the certainty of evidence, or pool effect estimates. Nearly half of the clinical studies had no comparator, and differences in populations, interventions, and outcomes limited comparisons. Coding findings as beneficial, non-significant, or unfavourable also omitted information about effect size, precision, and clinical importance, particularly when clinical studies reported several outcomes and time points. Only 30% of the extracted data was independently reviewed by a second reviewer, leaving some possibility of classification error. Limited safety reporting, publication bias, and selective reporting further restricted conclusions about the balance of benefits and harms.

## 5. Conclusions

Publication activity on mind–body therapies for comorbidity and multimorbidity has increased, although the literature remains geographically concentrated and international collaboration is limited. Bibliometric patterns showed sustained interest in chronic pain, trauma-related and substance use disorders, mindfulness-based approaches, and biofeedback or neurofeedback. The evidence map also showed that many condition combinations were examined in only a small number of clinical studies, with considerable variation in study designs, comparators, and outcomes. Although mindfulness-based interventions were studied most frequently, the available evidence does not show that they are more effective than other mind–body therapies. Future research should use robust comparative designs, include broader populations and settings, standardise outcomes, and improve long-term and safety reporting.

## Figures and Tables

**Table 1:** Top 8 contributing authors in research on mind–body therapies for multimorbidity.

Author	Publications	Institution	Country
Ward, Lesley	6	Department of Social Work, Education and Community Wellbeing, Northumbria University; Department of Sport, Exercise and Rehabilitation, Northumbria University	United Kingdom
Bissell, Laura	6	British Wheel of Yoga Qualifications	United Kingdom
Wiley, Laura	6	York Trials Unit, Department of Health Sciences, University of York	United Kingdom
Howsam, Jenny	6	British Wheel of Yoga Qualifications	United Kingdom
Rapley, Tim	6	Department of Social Work, Education and Community Wellbeing, Northumbria University	United Kingdom
Rose, Fiona	5	York Trials Unit, Department of Health Sciences, University of York	United Kingdom
Vranceanu, Ana-Maria	4	Centre for Health Outcomes and Interdisciplinary Research (CHOIR), Department of Psychiatry, Massachusetts General Hospital; Department of Psychiatry, Harvard Medical School	United States
Sheikh, Mohammad Asif	4	School of Psychological Sciences, Christ University	India

**Table 2:** Top 12 institutions by publication frequency in research on mind–body therapies for multimorbidity.

Rank	Publications	Centrality	Institutions
1	7	0.02	University of California
2	7	0.01	Harvard University
3	6	0.01	Massachusetts General Hospital
4	5	0.03	Brown University
5	5	0	University of York
6	5	0	Northumbria University
7	4	0.01	University of Minnesota
8	4	0	York St John University
9	3	0.01	VA Greater Los Angeles Healthcare System
10	3	0	University of Toronto
10	3	0	VA Portland Health Care System
10	3	0	Christ University

**Table 3:** Most frequently references by citation frequency in research on mind–body therapies for multimorbidity.

Freq	Author	Title	Source
3	Laban-Sharman A, 2023 [18]	Systematic review to explore the effect of yoga on anxiety in adults	Mental Health: Global Challenges Journal
3	Davies JN, 2024 [19]	Prevalence and 20-year trends in meditation, yoga, guided imagery and progressive relaxation use among US adults from 2002 to 2022	Scientific Reports
3	Rapley T, 2021 [20]	Some Pragmatics of Qualitative Data Analysis	Qualitative Research (5th ed.)
3	Lu JY, 2024 [21]	The impact of Yoga on patients with knee osteoarthritis: A systematic review and meta-analysis of randomised controlled trials	PLoS ONE
3	Bricca A, 2020 [22]	Benefits and harms of exercise therapy in people with multimorbidity: A systematic review and meta-analysis of randomised controlled trials	Ageing Research Reviews
3	Tew GA, 2021 [23]	Yoga for older adults with multimorbidity (the Gentle Years Yoga Trial): Study protocol for a randomised controlled trial	Trials
3	Anheyer D, 2022 [24]	Yoga for treating low back pain: A systematic review and meta-analysis	PAIN

## Data Availability

The raw data supporting the conclusions of this article will be made available by the authors on request.

## Supplementary Materials

The following supporting information can be downloaded at https://www.mdpi.com/article/10.3390/healthcare14182902/s1, Table S1. Item-by-item BIBLIO reporting checklist. Table S2. Database-specific search strategies. Table S3. Operational definitions and coding rules for study-level evidence mapping. Table S4. Study-level evidence-map dataset. Table S5. Study-design distribution by condition combination. Table S6. Study-level distribution of outcome domains and directions.
