# Supplementary material for: Mind–Body Therapies for Multimorbidity: A Bibliometric Analysis and Evidence Map
Source: Healthcare (Basel). 2026 Sep 8;14(18):2902. doi: 10.3390/healthcare14182902 (PMC13606127; doi:10.3390/healthcare14182902)
Supplement: Supplementary file 1 [file healthcare-14-02902-s001.zip › healthcare-4444538-supplementary.pdf]

Supplementary material

Table S1. Item-by-item BIBLIO reporting checklist.

Section/Topic	Item No.	Checklist item	Reported on page No.
**Title**			
Identification	1	Identify the report as a bibliometric review in the title.	1
Issues/topics	2	Indicate the key issues/topics under investigation and coverage of time period.	1
**Abstract**			
Structured summary	3	Structured summary including (as applicable): background, methods, results (key findings) and conclusions.	1-2
**Introduction/ Background**			
Justification/ Rationale/ Explanation	4	Present review of existing knowledge and epidemiological information.	2-3
Objectives	5	Statement of the objective (s) or question (s).	3-4
**Methods**			
Search engines (data sources)	6	Describe all information sources (such as electronic databases, contact with study authors, trial registers or other grey literature sources).	4
Search strategy	7	Keywords and systematization criteria (date of search, language, type of document) for the search.	4
Time period	8	The period that the review covers and the justification.	4
Eligibility criteria	9	Describe all inclusion and exclusion criteria; languages; study design, type of publication and time period.	4
Data refinement (data selection procedure)	10	Remove the irrelevant articles; inspection to eliminate duplicate and unrelated articles (after evaluation of the title, abstract and content).	7
Quality assessment (optional)	11	Assessment of papers by three authors and the use of assessing checklists.	4
Data synthesis	12	Describe the methods used for summarizing, handling, synthesis, tabulations or schematic displays. Describe how the data were analysed.	5-6
**Results**			
Descriptive findings (statistics)	13	- Provide details of the search and selection process in a flow diagram.<br>- Number of citations retrieved (number of publication, year of	7-18

		publication, type of documents, country of publication, articles with the highest impact, most impactful authors, most impactful articles, authors with the highest production, top journals, top institutions, …)	
Schematic map and trend	14	Summarize and/or present the schematic maps and trends using an appropriate software to present citations, journals, authors, top journals, time trends, emerging literature, and any relevant indicators (as applicable).	7-18
Tabulation and summarizing the findings	15	General recommendation: Studies under consideration could be summarized and organized by different subtitles and different scenarios. Regardless, results need to be presented in separate tables covering each subtitle. The followings are some options that could help to summarize the findings.<br><br>*Option 1:*<br>- Start the presentation with a historical view [when and who first published on the topic].<br>- Report on review papers. The result should be listed in a separate table. Also, specify the review type (scoping review, narrative review, systematic review, and meta-analysis).<br>- Summarize the findings according to the study designs and main study types.<br>*Option 2:*<br>- Start the presentation with a historical view [when and who first published on the topic].<br>- Report on review papers. The result should be listed in a separate table. Also, indicate the review type (scoping review, narrative review, systematic review, and meta-analysis) should be specified.<br>- Summarize the findings according to outcome measures or populations. .<br>*Option 3:*<br>- Start the presentation with a historical view [when and who first published on the topic].<br>- Report on review papers. The result should be listed in a separate table. Also, specify the review type (scoping review, narrative review, systematic review, and meta-analysis).<br>- Summarize the findings according to concept.<br>*Option 4.*<br>- Start the presentation with a historical view [when and who first published on the topic].<br>- Report on review papers. The result should be listed in a separate table, and also specify the review type (scoping review, narrative review, systematic review, and meta-analysis).<br>- Summarize the findings according to different subtitles relevant to the main topic .	7-18
Synthesis of findings	16	Synthesize the findings as much as possible, find the gap, and	-

		propose a model, hypothesis, etc. (if applicable).	
**Discussion**			
Summary of evidence	17	Summarize the main findings. The findings should be presented in more "general" or "accessible" terms.	19-21
Interpretation	18	Include interpretation consistent with results. Explanations for observed outcomes, similarities, and differences reported would be essential.	19-21
Strengths and limitations	19	Discuss the strengths and limitations.	24
Conclusion(s)	20	Provide a general interpretation of the results with respect to the review questions and objectives, as well as potential implications.	25

Source: Adapted from Montazeri et al. (2023), cited as Ref. [12] in the main manuscript, under the Creative Commons Attribution 4.0 International License.

Table S2. Database-specific search strategies.

Database	Retrieval formula	Results (2026-07-30)
Web of Science Core Collection (WoSCC)	#1 TS = ("mind-body therap*" OR "mind body therap*" OR "mind-body medicine" OR "mind body medicine" OR "mind-body intervention*" OR "mind body intervention*" OR "mind-body practice*" OR "mind body practice*" OR "mind-body exercise*" OR "mind body exercise*" OR meditation OR mindfulness OR "mindfulness-based" OR "mindfulness based" OR "mindfulness-based cognitive therap*" OR "mindfulness based cognitive therap*" OR "mindfulness-based stress reduction" OR "mindfulness based stress reduction" OR yoga OR "tai chi" OR taichi OR taijiquan OR qigong OR "qi gong" OR baduanjin OR "ba duan jin" OR "eight-section brocade" OR "eight section brocade" OR yijinjing OR "yi jin jing" OR "muscle tendon changing exercise" OR wuqinxi OR "wu qin xi" OR "five-animal exercise*" OR "five animal exercise*" OR liuzijue OR "liu zi jue" OR "six healing sounds" OR "relaxation therap*" OR "guided imagery" OR "progressive muscle relaxation" OR "progressive muscular relaxation" OR (PMR NEAR/3 relax*) OR "autogenic training" OR biofeedback OR neurofeedback OR hypnosis OR hypnotherapy)<br>#2 TS = (comorbid* OR co-morbid* OR multimorbid* OR multi-morbid* OR multidisease* OR multi-disease* OR multipatholog* OR multi-patholog* OR polypatholog* OR polymorbid* OR poly-morbid* OR pluripatholog* OR "multiple chronic condition*" OR "multiple chronic disease*" OR "multiple long-term condition*" OR "multiple health condition*" OR "coexisting chronic condition*" OR "co-existing chronic condition*" OR "co-occurring chronic condition*" OR "coexisting chronic disease*" OR "co-existing chronic disease*" OR "co-occurring chronic disease*)<br>#3 DOP=2004-01-01/2026-07-30<br>#4 #1 and #2 and #3<br>#5 #4 AND LA=(English) AND DT=(Article OR Review)	1,067
PubMed (MEDLINE)	#1 ("mind-body therap*"[Title/Abstract] OR "mind body therap*"[Title/Abstract] OR "mind-body medicine"[Title/Abstract] OR "mind body medicine"[Title/Abstract] OR "mind-body intervention*"[Title/Abstract] OR "mind body intervention*"[Title/Abstract] OR "mind-body practice*"[Title/Abstract] OR "mind body practice*"[Title/Abstract] OR "mind-body exercise*"[Title/Abstract] OR "mind body exercise*"[Title/Abstract] OR meditation[Title/Abstract] OR mindfulness[Title/Abstract] OR "mindfulness-based"[Title/Abstract] OR "mindfulness based"[Title/Abstract] OR "mindfulness-based	1,395

cognitive therap*"[Title/Abstract] OR "mindfulness based cognitive therap*"[Title/Abstract] OR "mindfulness-based stress reduction"[Title/Abstract] OR "mindfulness based stress reduction"[Title/Abstract] OR yoga[Title/Abstract] OR "tai chi"[Title/Abstract] OR taichi[Title/Abstract] OR taijiquan[Title/Abstract] OR qigong[Title/Abstract] OR "qi gong"[Title/Abstract] OR baduanjin[Title/Abstract] OR "ba duan jin"[Title/Abstract] OR "eight-section brocade"[Title/Abstract] OR "eight section brocade"[Title/Abstract] OR yijinjing[Title/Abstract] OR "yi jin jing"[Title/Abstract] OR "muscle tendon changing exercise"[Title/Abstract] OR wuqinxi[Title/Abstract] OR "wu qin xi"[Title/Abstract] OR "five-animal exercise*"[Title/Abstract] OR "five animal exercise*"[Title/Abstract] OR liuzijue[Title/Abstract] OR "liu zi jue"[Title/Abstract] OR "six healing sounds"[Title/Abstract] OR "relaxation therap*"[Title/Abstract] OR "guided imagery"[Title/Abstract] OR "progressive muscle relaxation"[Title/Abstract] OR "progressive muscular relaxation"[Title/Abstract]) OR ((PMR[Title/Abstract] OR PMRT[Title/Abstract]) AND relax*[Title/Abstract]) OR "autogenic training"[Title/Abstract] OR biofeedback[Title/Abstract] OR neurofeedback[Title/Abstract] OR hypnosis[Title/Abstract] OR hypnotherapy[Title/Abstract] OR "Meditation"[MeSH Terms] OR "Mindfulness"[MeSH Terms] OR "Yoga"[MeSH Terms] OR "Tai Ji"[MeSH Terms] OR "Qigong"[MeSH Terms] OR "Relaxation Therapy"[MeSH Terms] OR "Biofeedback, Psychology"[MeSH Terms] OR "Neurofeedback"[MeSH Terms] OR "Hypnosis"[MeSH Terms]）

#2 comorbid*[Title/Abstract] OR co-morbid*[Title/Abstract] OR multimorbid*[Title/Abstract] OR multi-morbid*[Title/Abstract] OR multidisease*[Title/Abstract] OR multi-disease*[Title/Abstract] OR multipatholog*[Title/Abstract] OR multi-patholog*[Title/Abstract] OR polypatholog*[Title/Abstract] OR polymorbid*[Title/Abstract] OR poly-morbid*[Title/Abstract] OR pluripatholog*[Title/Abstract] OR "multiple chronic condition*"[Title/Abstract] OR "multiple chronic disease*"[Title/Abstract] OR "multiple long-term condition*"[Title/Abstract] OR "multiple health condition*"[Title/Abstract] OR "coexisting chronic condition*"[Title/Abstract] OR "co-existing chronic condition*"[Title/Abstract] OR "co-occurring chronic condition*"[Title/Abstract] OR "coexisting chronic disease*"[Title/Abstract] OR "co-existing chronic disease*"[Title/Abstract] OR "co-occurring chronic disease*"[Title/Abstract] OR "Comorbidity"[MeSH Terms] OR "Multimorbidity"[MeSH Terms]

#3 "2004/01/01"[Date - Publication] : "2026/07/30"[Date -

	Publication]<br>#4 #1 and #2 and #3	
Embase	#1 'mind body therapy'/exp OR 'mind body therapy' OR 'mind body medicine'/exp OR 'mind body medicine' OR 'meditation'/exp OR 'meditation' OR 'mindfulness'/exp OR 'mindfulness' OR 'mindfulness based cognitive therapy'/exp OR 'mindfulness based cognitive therapy' OR 'mindfulness based stress reduction'/exp OR 'mindfulness based stress reduction' OR 'yoga'/exp OR 'yoga' OR 'tai chi'/exp OR 'tai chi' OR 'qigong'/exp OR 'qigong' OR 'relaxation training'/exp OR 'relaxation training' OR 'guided imagery'/exp OR 'guided imagery' OR 'autogenic training'/exp OR 'autogenic training' OR 'biofeedback'/exp OR 'biofeedback' OR 'neurofeedback'/exp OR 'neurofeedback' OR 'hypnosis'/exp OR 'hypnosis' OR 'hypnotherapy'/exp OR 'hypnotherapy' OR 'mind-body therap*':ti,ab,kw OR 'mind body therap*':ti,ab,kw OR 'mind-body medicine':ti,ab,kw OR 'mind body medicine':ti,ab,kw OR 'mind-body intervention*':ti,ab,kw OR 'mind body intervention*':ti,ab,kw OR 'mind-body practice*':ti,ab,kw OR 'mind body practice*':ti,ab,kw OR 'mind-body exercise*':ti,ab,kw OR 'mind body exercise*':ti,ab,kw OR meditation:ti,ab,kw OR mindfulness:ti,ab,kw OR 'mindfulness-based':ti,ab,kw OR 'mindfulness based':ti,ab,kw OR 'mindfulness-based cognitive therap*':ti,ab,kw OR 'mindfulness based cognitive therap*':ti,ab,kw OR 'mindfulness-based stress reduction':ti,ab,kw OR 'mindfulness based stress reduction':ti,ab,kw OR yoga:ti,ab,kw OR 'tai chi':ti,ab,kw OR taichi:ti,ab,kw OR taijiquan:ti,ab,kw OR qigong:ti,ab,kw OR 'qi gong':ti,ab,kw OR baduanjin:ti,ab,kw OR 'ba duan jin':ti,ab,kw OR 'eight-section brocade':ti,ab,kw OR 'eight section brocade':ti,ab,kw OR yijinjing:ti,ab,kw OR 'yi jin jing':ti,ab,kw OR 'muscle tendon changing exercise':ti,ab,kw OR wuqinxi:ti,ab,kw OR 'wu qin xi':ti,ab,kw OR 'five-animal exercise*':ti,ab,kw OR 'five animal exercise*':ti,ab,kw OR liuzijue:ti,ab,kw OR 'liu zi jue':ti,ab,kw OR 'six healing sounds':ti,ab,kw OR 'relaxation therap*':ti,ab,kw OR 'guided imagery':ti,ab,kw OR 'progressive muscle relaxation':ti,ab,kw OR 'progressive muscular relaxation':ti,ab,kw OR (((pmr OR pmrt) NEAR/3 relax*):ti,ab,kw) OR 'autogenic training':ti,ab,kw OR biofeedback:ti,ab,kw OR neurofeedback:ti,ab,kw OR hypnosis:ti,ab,kw OR hypnotherapy:ti,ab,kw<br>#2 'comorbidity'/exp OR 'comorbidity' OR 'multimorbidity'/exp OR 'multimorbidity' OR 'multiple chronic conditions'/exp OR 'multiple chronic conditions' OR comorbid*:ti,ab,kw OR 'co morbid*':ti,ab,kw OR multimorbid*:ti,ab,kw OR 'multi morbid*':ti,ab,kw OR multidisease*:ti,ab,kw OR 'multi disease*':ti,ab,kw OR multipatholog*:ti,ab,kw OR 'multi patholog*':ti,ab,kw OR polypatholog*:ti,ab,kw OR polymorbid*:ti,ab,kw OR 'poly	3,979

	morbid*':ti,ab,kw OR pluripatholog*:ti,ab,kw OR 'multiple chronic condition*':ti,ab,kw OR 'multiple chronic disease*':ti,ab,kw OR 'multiple long-term condition*':ti,ab,kw OR 'multiple long term condition*':ti,ab,kw OR 'multiple health condition*':ti,ab,kw OR 'coexisting chronic condition*':ti,ab,kw OR 'co-existing chronic condition*':ti,ab,kw OR 'co-occurring chronic condition*':ti,ab,kw OR 'cooccurring chronic condition*':ti,ab,kw OR 'coexisting chronic disease*':ti,ab,kw OR 'co-existing chronic disease*':ti,ab,kw OR 'co-occurring chronic disease*':ti,ab,kw OR 'cooccurring chronic disease*':ti,ab,kw<br>#3 [01-01-2004]/sd NOT [30-07-2026]/sd<br>#4 #1 and #2 and #3<br>#5 #4 AND [english]/lim AND ([article]/lim OR [review]/lim)

Table S3. Operational definitions and coding rules for study-level evidence mapping

Categories and operational rules used for study-level evidence mapping (n = 77).

Section	Category	Operational definition
Broad pattern	Mental–mental	Both coexisting conditions classified as mental, behavioural or neurodevelopmental disorders.
Broad pattern	Mental–physical	One condition classified as mental, behavioural or neurodevelopmental; the other as physical.
Broad pattern	Physical–physical	Both coexisting conditions classified as physical disorders.
Broad pattern	Unspecified multimorbidity	The report identified multimorbidity but did not permit a specific condition pair to be assigned.
Intervention	Mindfulness-based interventions	Explicit mindfulness training, including mindfulness-based cognitive therapy or mindfulness-based stress reduction.
Intervention	Multicomponent mind-body interventions	Two or more eligible modalities, or an eligible modality plus a structured component when no modality predominated.
Intervention	Biofeedback-based therapies	Biofeedback or neurofeedback was the defining active component.
Intervention	Yoga	Yoga was the defining active modality.
Intervention	General mind-body interventions	The intervention was described too broadly for assignment to a specific modality.
Intervention	Meditation	Meditation was the defining modality without a more specific mindfulness classification.
Intervention	Tai chi	Tai chi or qigong was the defining modality.
Intervention	Hypnosis	Hypnosis was the defining modality.
Intervention	Relaxation techniques	Relaxation or breathing techniques were the defining modality.
Comparator	No comparator	No concurrent clinical comparator was reported.
Comparator	Usual care	Routine care or treatment as usual.
Comparator	Active mind-body intervention	A different eligible mind-body intervention served as comparator.
Comparator	Active treatment control	A non-mind-body active treatment or behavioural intervention served as comparator.
Comparator	Attention or education control	Attention-matched, education, or health-information control.
Comparator	Wait-list or delayed intervention	Wait-list, delayed-treatment, or deferred-intervention control.
Comparator	Multiple comparator types	More than one comparator category was used.
Comparator	Pharmacological treatment	Medication or pharmacological treatment served as comparator.
Comparator	Sham or placebo control	Sham, placebo, or non-contingent feedback control.
Outcome domain	Psychological outcomes	Depression, anxiety, distress, cognition, or related psychological measures.
Outcome domain	Clinical symptoms	Disease symptoms, pain, fatigue, or other clinical symptom measures.
Outcome domain	Healthcare utilisation or economic outcomes	Healthcare use, costs, resource use, or economic outcomes.
Outcome domain	Physiological or biomarker outcomes	Physiological measures, laboratory markers, or biomarkers.
Outcome domain	Physical functioning	Physical performance, disability, activity, or functional status.
Outcome domain	Health-related quality of life	Health-related quality-of-life measures.
Outcome	Subjective well-being	Well-being, life satisfaction, or related self-reported measures.

domain		
Outcome domain	Sleep	Sleep quality, insomnia, or sleep-related measures.
Outcome domain	Social relationships	Social functioning, interpersonal relationships, or social participation.
Direction	Beneficial	At least one reported outcome favoured the intervention or showed improvement.
Direction	Non-significant	The reported comparison or change was not statistically significant.
Direction	Unfavourable	At least one reported outcome favoured the comparator or showed worsening.
Direction	Favourable safety finding	No safety concern or a favourable safety outcome was reported.
Direction	Safety concern	A clinical symptom or other safety concern was reported.
Study design	Randomised controlled trial	Random allocation to intervention and comparator groups.
Study design	Case report or case series	One or more clinical cases without a controlled comparative design.
Study design	Single-arm intervention study	Intervention study without a concurrent comparator.
Study design	Secondary analysis of randomised trial	Secondary analysis using data from a completed randomised trial.
Study design	Mixed-methods intervention study	Intervention study combining quantitative and qualitative methods.
Study design	Non-randomised controlled trial	Controlled intervention study without random allocation.
Study design	Observational study	Observational clinical evaluation that included delivery of an eligible intervention.
Study design	Secondary or exploratory analysis	Intervention-related secondary or exploratory analysis not derived from a completed randomised trial.
ICD-11 rule	Disease-system combination	Each named condition was mapped to WHO ICD-11 MMS release 2026-01; pairs were treated as nondirectional and each study was counted once.

Note: Conventional cognitive behavioural therapy alone was not classified as an eligible mind-body therapy. WHO ICD-11 source: https://icd.who.int/browse/2026-01/mms/en

Table S4. Study-level evidence-map dataset

One row per unique clinical study; outcome domains and directions are semicolon-separated and are not mutually exclusive.

Study ID	Study design	Condition combination	Broad pattern	Intervention	Comparator	ICD-11 system combination	Outcome domains	Outcome directions
001	Randomised controlled trial	Obsessive-compulsive disorder + Depressive disorder	Mental-mental comorbidity	Mindfulness-based interventions	Active treatment control	Mental/behavioural disorders + Mental/behavioural disorders	Clinical symptoms; Healthcare utilisation and economic outcomes; Psychological outcomes; Subjective well-being	Favourable safety finding; Non-significant; Beneficial
002	Case report or case series	Insomnia disorder + Chronic pain	Mental-physical comorbidity	Multicomponent mind-body interventions	No comparator	Mental/behavioural disorders + Neurological/pain disorders	Clinical symptoms; Physical functioning; Physiological or biomarker outcomes	Beneficial; Unfavourable
003	Case report or case series	Pangastritis + Meniere disease	Physical-physical comorbidity	Multicomponent mind-body interventions	No comparator	Gastrointestinal disorders + Neurological/pain disorders	Clinical symptoms; Health-related quality of life; Physiological or biomarker outcomes; Psychological outcomes	Beneficial; Favourable safety finding; Unfavourable
004	Non-randomised controlled trial	Internet gaming disorder + Depressive disorder	Mental-mental comorbidity	Mindfulness-based interventions	Active mind-body intervention	Mental/behavioural disorders + Mental/behavioural disorders	Physiological or biomarker outcomes; Psychological outcomes	Beneficial; Non-significant
005	Single-arm intervention study	Obsessive-compulsive disorder + Major depressive disorder	Mental-mental comorbidity	Mindfulness-based interventions	No comparator	Mental/behavioural disorders + Mental/behavioural disorders	Psychological outcomes; Subjective well-being	Beneficial
006	Single-arm intervention study	Post-traumatic stress disorder + Substance use disorder	Mental-mental comorbidity	Mindfulness-based interventions	No comparator	Mental/behavioural disorders + Mental/behavioural disorders	Clinical symptoms; Health-related quality of life; Healthcare utilisation and economic outcomes; Psychological outcomes	Beneficial; Non-significant; Unfavourable

007	Case report or case series	Cyclic vomiting syndrome + Superior mesenteric artery syndrome	Physical-physical comorbidity	Multicomponent mind-body interventions	No comparator	Gastrointestinal disorders + Gastrointestinal disorders	Clinical symptoms; Healthcare utilisation and economic outcomes; Physiological or biomarker outcomes; Psychological outcomes	Beneficial; Non-significant; Unfavourable
008	Randomised controlled trial	Multimorbidity + Multimorbidity	Unspecified multimorbidity	Yoga	Usual care	Multimorbidity	Clinical symptoms; Health-related quality of life; Healthcare utilisation and economic outcomes; Physical functioning; Psychological outcomes; Sleep; Social relationships	Beneficial; Non-significant; Favourable safety finding; Unfavourable
009	Single-arm intervention study	HIV infection + Depressive disorder	Mental-physical comorbidity	Multicomponent mind-body interventions	No comparator	Infectious/immune/dermatologic disorders + Mental/behavioural disorders	Health-related quality of life; Healthcare utilisation and economic outcomes; Psychological outcomes	Non-significant; Beneficial
010	Single-arm intervention study	Chronic musculoskeletal pain + Alzheimer disease	Physical-physical comorbidity	Yoga	No comparator	Neurological/pain disorders + Neurological/pain disorders	Clinical symptoms; Health-related quality of life; Healthcare utilisation and economic outcomes; Psychological outcomes; Sleep	Beneficial; Non-significant; Favourable safety finding; Unfavourable
011	Randomised controlled trial	Attention-deficit/hyperactivity disorder + Oppositional defiant disorder	Mental-mental comorbidity	Multicomponent mind-body interventions	Multiple comparator types	Mental/behavioural disorders + Mental/behavioural disorders	Psychological outcomes	Beneficial; Non-significant
012	Secondary analysis of randomized	Attention-deficit/hyperactivity disorder +	Mental-mental comorbidity	Biofeedback-based therapies	Sham or placebo control	Mental/behavioural disorders + Mental/behavioural	Healthcare utilisation and	Beneficial; Non-significant; Unfavourable

	trial	Oppositional defiant disorder				disorders	economic outcomes; Psychological outcomes	
013	Case report or case series	Type 2 diabetes + COVID-19	Physical-physical comorbidity	Multicomponent mind-body interventions	No comparator	Cardiometabolic/endocrine disorders + Infectious/immune/dermatologic disorders	Clinical symptoms; Health-related quality of life; Physiological or biomarker outcomes	Beneficial
014	Mixed-methods intervention study	Post-traumatic stress disorder + Depressive disorder	Mental-mental comorbidity	Mindfulness-based interventions	No comparator	Mental/behavioural disorders + Mental/behavioural disorders	Healthcare utilisation and economic outcomes; Psychological outcomes	Beneficial; Unfavourable
015	Case report or case series	Type 2 diabetes + COVID-19	Physical-physical comorbidity	Multicomponent mind-body interventions	No comparator	Cardiometabolic/endocrine disorders + Infectious/immune/dermatologic disorders	Clinical symptoms; Physiological or biomarker outcomes; Psychological outcomes	Beneficial
016	Single-arm intervention study	Post-traumatic stress disorder + Chronic pain	Mental-physical comorbidity	Yoga	No comparator	Mental/behavioural disorders + Neurological/pain disorders	Clinical symptoms; Healthcare utilisation and economic outcomes; Physical functioning; Psychological outcomes; Social relationships	Non-significant; Beneficial; Unfavourable
017	Case report or case series	Type 2 diabetes + Pemphigus vulgaris	Physical-physical comorbidity	Multicomponent mind-body interventions	No comparator	Cardiometabolic/endocrine disorders + Infectious/immune/dermatologic disorders	Clinical symptoms; Health-related quality of life; Healthcare utilisation and economic outcomes; Physiological or biomarker outcomes	Non-significant; Beneficial
018	Secondary analysis of randomized trial	Asthma + Panic disorder	Mental-physical comorbidity	Biofeedback-based therapies	Active mind-body intervention	Mental/behavioural disorders + Respiratory/ENT disorders	Clinical symptoms; Physiological or biomarker	Non-significant

							outcomes; Psychological outcomes	
019	Randomised controlled trial	Cardiovascular disease + Depressive disorder	Mental-physical comorbidity	Mindfulness-based interventions	Multiple comparator types	Cardiometabolic/endocrine disorders + Mental/behavioural disorders	Clinical symptoms; Health-related quality of life; Healthcare utilisation and economic outcomes; Physiological or biomarker outcomes; Psychological outcomes; Subjective well-being	Non-significant; Beneficial; Unfavourable
020	Secondary analysis of randomized trial	Chronic pain + Depressive disorder	Mental-physical comorbidity	Mindfulness-based interventions	Usual care	Mental/behavioural disorders + Neurological/pain disorders	Psychological outcomes	Beneficial; Non-significant
021	Randomised controlled trial	Post-traumatic stress disorder + Substance use disorder	Mental-mental comorbidity	Mindfulness-based interventions	Multiple comparator types	Mental/behavioural disorders + Mental/behavioural disorders	Clinical symptoms; Psychological outcomes; Subjective well-being	Beneficial; Non-significant
022	Case report or case series	Asthma + Vocal cord dysfunction	Physical-physical comorbidity	Hypnosis	No comparator	Respiratory/ENT disorders + Respiratory/ENT disorders	Clinical symptoms; Healthcare utilisation and economic outcomes; Sleep	Beneficial
023	Randomised controlled trial	Post-traumatic stress disorder + Bipolar disorder	Mental-mental comorbidity	Meditation	Usual care	Mental/behavioural disorders + Mental/behavioural disorders	Psychological outcomes	Beneficial
024	Randomised controlled trial	Post-traumatic stress disorder + Depressive disorder	Mental-mental comorbidity	General mind-body interventions	Active mind-body intervention	Mental/behavioural disorders + Mental/behavioural disorders	Clinical symptoms; Healthcare utilisation and economic outcomes; Psychological outcomes	Beneficial; Non-significant; Unfavourable
025	Case report or case series	Depressive disorder + Eating disorder	Mental-mental comorbidity	Meditation	No comparator	Mental/behavioural disorders + Mental/behavioural disorders	Clinical symptoms; Healthcare utilisation and economic outcomes; Psychologica	Beneficial; Non-significant

							l outcomes	
026	Single-arm intervention study	Binge-eating disorder + Substance use disorder	Mental-mental comorbidity	Mindfulness-based interventions	No comparator	Mental/behavioural disorders + Mental/behavioural disorders	Clinical symptoms; Healthcare utilisation and economic outcomes; Psychological outcomes	Beneficial; Non-significant; Unfavourable
027	Case report or case series	Obsessive-compulsive disorder + Depressive disorder	Mental-mental comorbidity	Mindfulness-based interventions	No comparator	Mental/behavioural disorders + Mental/behavioural disorders	Healthcare utilisation and economic outcomes; Psychological outcomes; Subjective well-being	Beneficial; Unfavourable
028	Observational study	Type 2 diabetes + Hypertension	Physical-physical comorbidity	Yoga	No comparator	Cardiometabolic/endocrine disorders + Cardiometabolic/endocrine disorders	Physiological or biomarker outcomes	Beneficial; Non-significant
029	Case report or case series	Type 2 diabetes + Ehlers-Danlos syndrome	Physical-physical comorbidity	Multicomponent mind-body interventions	No comparator	Cardiometabolic/endocrine disorders + Musculoskeletal/connective tissue disorders	Clinical symptoms; Physical functioning; Physiological or biomarker outcomes; Psychological outcomes; Subjective well-being	Beneficial; Unfavourable; Non-significant
030	Randomised controlled trial	Post-traumatic stress disorder + Depressive disorder	Mental-mental comorbidity	Mindfulness-based interventions	Usual care	Mental/behavioural disorders + Mental/behavioural disorders	Health-related quality of life; Healthcare utilisation and economic outcomes; Psychological outcomes	Beneficial; Non-significant
031	Single-arm intervention study	Multimorbidity + Multimorbidity	Unspecified multimorbidity	Mindfulness-based interventions	No comparator	Multimorbidity	Healthcare utilisation and economic outcomes; Psychological outcomes; Subjective well-being	Unfavourable; Beneficial; Non-significant
032	Secondary or exploratory analysis	Multimorbidity + Multimorbidity	Unspecified multimorbidity	Biofeedback-based therapies	No comparator	Multimorbidity	Clinical symptoms; Physiological or biomarker outcomes;	Beneficial; Non-significant

							Psychological outcomes; Sleep	
033	Case report or case series	Agoraphobia + Panic disorder	Mental-mental comorbidity	Biofeedback-based therapies	No comparator	Mental/behavioural disorders + Mental/behavioural disorders	Healthcare utilisation and economic outcomes; Physiological or biomarker outcomes; Psychological outcomes	Beneficial; Non-significant; Unfavourable
034	Single-arm intervention study	Bipolar disorder + Depressive disorder	Mental-mental comorbidity	Multicomponent mind-body interventions	No comparator	Mental/behavioural disorders + Mental/behavioural disorders	Clinical symptoms; Health-related quality of life; Healthcare utilisation and economic outcomes; Psychological outcomes	Beneficial; Unfavourable; Non-significant
035	Single-arm intervention study	Multimorbidity + Multimorbidity	Unspecified multimorbidity	Multicomponent mind-body interventions	No comparator	Multimorbidity	Clinical symptoms; Healthcare utilisation and economic outcomes; Physiological or biomarker outcomes; Psychological outcomes	Beneficial
036	Randomised controlled trial	HIV infection + Depressive disorder	Mental-physical comorbidity	Multicomponent mind-body interventions	Active mind-body intervention	Infectious/immune/dermatologic disorders + Mental/behavioural disorders	Physiological or biomarker outcomes; Psychological outcomes	Beneficial
037	Observational study	Multimorbidity + Multimorbidity	Unspecified multimorbidity	Mindfulness-based interventions	No comparator	Multimorbidity	Physical functioning; Psychological outcomes	Beneficial
038	Randomised controlled trial	Depressive disorder + Drug dependence	Mental-mental comorbidity	Mindfulness-based interventions	Pharmacological treatment	Mental/behavioural disorders + Mental/behavioural disorders	Psychological outcomes	Beneficial
039	Randomised controlled trial	Personality disorder + Panic disorder	Mental-mental comorbidity	Mindfulness-based interventions	No comparator	Mental/behavioural disorders + Mental/behavioural disorders	Healthcare utilisation and economic outcomes; Psychological outcomes	Unfavourable; Beneficial

040	Randomised controlled trial	Depressive disorder + Anxiety disorder	Mental-mental comorbidity	Mindfulness-based interventions	Wait-list or delayed intervention	Mental/behavioural disorders + Mental/behavioural disorders	Psychological outcomes; Subjective well-being	Beneficial
041	Randomised controlled trial	Depressive disorder + Inflammatory bowel disease	Mental-physical comorbidity	Mindfulness-based interventions	Usual care	Gastrointestinal disorders + Mental/behavioural disorders	Health-related quality of life; Healthcare utilisation and economic outcomes; Physiological or biomarker outcomes; Psychological outcomes	Non-significant; Beneficial
042	Case report or case series	Post-traumatic stress disorder + Specific phobia	Mental-mental comorbidity	General mind-body interventions	No comparator	Mental/behavioural disorders + Mental/behavioural disorders	Clinical symptoms; Psychological outcomes; Sleep	Favourable safety finding; Beneficial
043	Mixed-methods intervention study	Multimorbidity + Multimorbidity	Unspecified multimorbidity	Multicomponent mind-body interventions	No comparator	Multimorbidity	Clinical symptoms; Healthcare utilisation and economic outcomes; Physical functioning; Psychological outcomes; Social relationships; Subjective well-being	Unsafe; Beneficial
044	Randomised controlled trial	Multimorbidity + Multimorbidity	Unspecified multimorbidity	Meditation	Attention or education control	Multimorbidity	Clinical symptoms; Physiological or biomarker outcomes; Psychological outcomes; Sleep; Subjective well-being	Beneficial; Non-significant
045	Randomised controlled trial	Multimorbidity + Multimorbidity	Unspecified multimorbidity	Tai chi	Attention or education control	Multimorbidity	Clinical symptoms; Physical functioning; Psychological outcomes	Non-significant; Unfavourable
046	Randomised controlled trial	Multimorbidity + Multimorbidity	Unspecified multimorbidity	Yoga	Usual care	Multimorbidity	Clinical symptoms; Health-related quality of life;	Beneficial; Favourable safety finding; Non-significant

							Healthcare utilisation and economic outcomes; Physical functioning; Psychological outcomes; Social relationships	
047	Single-arm intervention study	Multimorbidity + Multimorbidity	Unspecified multimorbidity	Tai chi	No comparator	Multimorbidity	Health-related quality of life; Healthcare utilisation and economic outcomes; Physical functioning; Physiological or biomarker outcomes	Beneficial; Non-significant; Unfavourable
048	Single-arm intervention study	Insomnia disorder + Depressive symptoms	Mental-mental comorbidity	Multicomponent mind-body interventions	No comparator	Mental/behavioural disorders + Mental/behavioural disorders	Healthcare utilisation and economic outcomes; Psychological outcomes; Sleep	Beneficial
049	Single-arm intervention study	Post-traumatic stress disorder + Irritable bowel syndrome	Mental-physical comorbidity	Mindfulness-based interventions	No comparator	Gastrointestinal disorders + Mental/behavioural disorders	Clinical symptoms; Psychological outcomes	Beneficial
050	Randomised controlled trial	Post-traumatic stress disorder + Substance use disorder	Mental-mental comorbidity	Mindfulness-based interventions	Active mind-body intervention	Mental/behavioural disorders + Mental/behavioural disorders	Healthcare utilisation and economic outcomes; Psychological outcomes	Beneficial; Unfavourable; Non-significant
051	Randomised controlled trial	Depressive disorder + Hwabyung	Mental-mental comorbidity	Multicomponent mind-body interventions	Wait-list or delayed intervention	Mental/behavioural disorders + Mental/behavioural disorders	Clinical symptoms; Psychological outcomes; Subjective well-being	Beneficial
052	Randomised controlled trial	Multimorbidity + Multimorbidity	Unspecified multimorbidity	Mindfulness-based interventions	Usual care	Multimorbidity	Clinical symptoms; Physical functioning; Psychological outcomes; Sleep; Social relationships	Beneficial; Non-significant
053	Secondary analysis of	Post-traumatic stress disorder +	Mental-mental comorbidity	Mindfulness-based	Active treatment	Mental/behavioural disorders +	Healthcare utilisation	Beneficial; Unfavourable

	randomized trial	Substance use disorder		interventions	control	Mental/behavioural disorders	and economic outcomes	
054	Non-randomised controlled trial	Attention-deficit/hyperactivity disorder + Idiopathic epilepsy	Mental-physical comorbidity	Biofeedback-based therapies	Pharmacological treatment	Mental/behavioural disorders + Neurological/pain disorders	Psychological outcomes	Beneficial; Non-significant
055	Case report or case series	Post-traumatic stress disorder + Chronic pain	Mental-physical comorbidity	Biofeedback-based therapies	No comparator	Mental/behavioural disorders + Neurological/pain disorders	Clinical symptoms; Healthcare utilisation and economic outcomes; Physical functioning; Psychological outcomes; Sleep	Beneficial; Non-significant
056	Secondary analysis of randomized trial	Post-traumatic stress disorder + Mild traumatic brain injury	Mental-physical comorbidity	Multicomponent mind-body interventions	Attention or education control	Mental/behavioural disorders + Neurological/pain disorders	Physical functioning; Psychological outcomes	Beneficial
057	Secondary analysis of randomized trial	Chronic musculoskeletal pain + Depressive disorder	Mental-physical comorbidity	General mind-body interventions	Usual care	Mental/behavioural disorders + Neurological/pain disorders	Clinical symptoms; Psychological outcomes	Beneficial
058	Randomised controlled trial	Post-traumatic stress disorder + Eating disorder	Mental-mental comorbidity	Biofeedback-based therapies	Sham or placebo control	Mental/behavioural disorders + Mental/behavioural disorders	Clinical symptoms; Healthcare utilisation and economic outcomes; Physiological or biomarker outcomes; Psychological outcomes; Subjective well-being	Beneficial; Non-significant
059	Single-arm intervention study	Post-traumatic stress disorder + Chronic pain	Mental-physical comorbidity	Yoga	No comparator	Mental/behavioural disorders + Neurological/pain disorders	Clinical symptoms; Healthcare utilisation and economic outcomes	Favourable safety finding; Beneficial; Unfavourable
060	Single-arm intervention study	Post-traumatic stress disorder + Chronic pain	Mental-physical comorbidity	Biofeedback-based therapies	No comparator	Mental/behavioural disorders + Neurological/pain disorders	Clinical symptoms; Healthcare utilisation and economic outcomes; Psychological outcomes; Sleep	Beneficial; Non-significant

061	Secondary analysis of randomized trial	Anxiety disorder + Alcohol use disorder	Mental-mental comorbidity	General mind-body interventions	Active mind-body intervention	Mental/behavioural disorders + Mental/behavioural disorders	Clinical symptoms	Beneficial; Non-significant
062	Secondary analysis of randomized trial	Post-traumatic stress disorder + Alcohol use disorder	Mental-mental comorbidity	General mind-body interventions	Active treatment control	Mental/behavioural disorders + Mental/behavioural disorders	Clinical symptoms; Healthcare utilisation and economic outcomes; Physiological or biomarker outcomes; Psychological outcomes	Non-significant; Unfavourable; Beneficial
063	Randomised controlled trial	Depressive disorder + Psychotic disorder	Mental-mental comorbidity	General mind-body interventions	Usual care	Mental/behavioural disorders + Mental/behavioural disorders	Healthcare utilisation and economic outcomes; Psychological outcomes; Social relationships	Beneficial; Non-significant
064	Mixed-methods intervention study	Coronary heart disease + Bipolar disorder	Mental-physical comorbidity	Mindfulness-based interventions	Usual care	Cardiometabolic/endocrine disorders + Mental/behavioural disorders	Clinical symptoms; Physiological or biomarker outcomes; Psychological outcomes	Non-significant; Beneficial; Unfavourable
065	Secondary analysis of randomized trial	Attention-deficit/hyperactivity disorder + Learning disorder	Mental-mental comorbidity	Multicomponent mind-body interventions	Wait-list or delayed intervention	Mental/behavioural disorders + Mental/behavioural disorders	Physiological or biomarker outcomes; Psychological outcomes	Beneficial; Non-significant
066	Secondary analysis of randomized trial	History of breast cancer + Insomnia disorder	Mental-physical comorbidity	Tai chi	Active treatment control	Mental/behavioural disorders + Neoplasms	Clinical symptoms; Physiological or biomarker outcomes; Psychological outcomes; Sleep	Non-significant; Beneficial; Unfavourable
067	Secondary or exploratory analysis	Chronic pain + Early cognitive decline	Physical-physical comorbidity	Multicomponent mind-body interventions	No comparator	Neurological/pain disorders + Neurological/pain disorders	Clinical symptoms; Physical functioning; Psychological outcomes; Subjective well-being	Beneficial; Non-significant
068	Randomised controlled trial	Awake bruxism + Chronic migraine	Physical-physical comorbidity	Biofeedback-based therapies	No comparator	Neurological/pain disorders + Neurological/pain disorders	Clinical symptoms	Beneficial; Favourable safety finding

069	Case report or case series	Attention-deficit/hyperactivity disorder + Post-traumatic stress disorder	Mental-mental comorbidity	Biofeedback-based therapies	No comparator	Mental/behavioural disorders + Mental/behavioural disorders	Clinical symptoms; Health-related quality of life; Healthcare utilisation and economic outcomes; Psychological outcomes; Social relationships	Beneficial; Favourable safety finding; Unsafe; Non-significant
070	Case report or case series	Generalized anxiety disorder + Hypothyroidism	Mental-physical comorbidity	Multicomponent mind-body interventions	No comparator	Cardiometabolic/endocrine disorders + Mental/behavioural disorders	Clinical symptoms; Physiological or biomarker outcomes; Psychological outcomes	Beneficial
071	Randomised controlled trial	Persistent postural-perceptual dizziness + Anxiety disorder	Mental-physical comorbidity	Multicomponent mind-body interventions	No comparator	Mental/behavioural disorders + Neurological/pain disorders	Clinical symptoms; Physiological or biomarker outcomes; Psychological outcomes; Social relationships	Non-significant; Beneficial
072	Secondary analysis of randomized trial	Chronic migraine + Medication-overuse headache	Physical-physical comorbidity	Mindfulness-based interventions	Usual care	Neurological/pain disorders + Neurological/pain disorders	Clinical symptoms; Healthcare utilisation and economic outcomes; Physiological or biomarker outcomes; Psychological outcomes	Beneficial; Non-significant; Favourable safety finding
073	Randomised controlled trial	Post-traumatic stress disorder + Chronic pain	Mental-physical comorbidity	Multicomponent mind-body interventions	Usual care	Mental/behavioural disorders + Neurological/pain disorders	Clinical symptoms; Physical functioning	Beneficial; Non-significant; Favourable safety finding
074	Randomised controlled trial	Migraine + Multiple sclerosis	Physical-physical comorbidity	Relaxation techniques	Attention or education control	Neurological/pain disorders + Neurological/pain disorders	Clinical symptoms; Healthcare utilisation and economic outcomes; Physical functioning	Non-significant; Beneficial; Unfavourable
075	Randomised controlled	Depressive symptoms +	Mental-physical	Yoga	Pharmacological treatment	Mental/behavioural disorders +	Physical functioning;	Beneficial; Non-significant

	trial	Rheumatoid arthritis	comorbidity			Musculoskeletal/connective tissue disorders	Physiological or biomarker outcomes; Psychological outcomes	
076	Randomised controlled trial	Chronic pain + Depressive disorder	Mental-physical comorbidity	Mindfulness-based interventions	Wait-list or delayed intervention	Mental/behavioural disorders + Neurological/pain disorders	Clinical symptoms; Health-related quality of life; Healthcare utilisation and economic outcomes; Psychological outcomes	Non-significant; Favourable safety finding; Beneficial; Unfavourable
077	Non-randomised controlled trial	Borderline personality disorder + Substance use disorder	Mental-mental comorbidity	Yoga	Active treatment control	Mental/behavioural disorders + Mental/behavioural disorders	Psychological outcomes	Beneficial; Non-significant

Note: Study-level counts use 77 unique clinical studies as the denominator. Outcome-direction labels are descriptive and are not pooled estimates of efficacy or safety

Table S5. Study-design distribution by condition combination

Each study is counted once within its specific condition combination; rows and design columns sum to 77 studies.

Code	Condition combination	Broad pattern	ICD-11 system combination	RCT	NRCT	Single-arm	Mixed-methods	Observational	Case/series	Secondary RCT	Secondary/exploratory	Total	%
C01	Multimorbidity + Multimorbidity	Unspecified multimorbidity	Multimorbidity	5	0	3	1	1	0	0	1	11	14.3%
C02	Chronic pain + Post-traumatic stress disorder	Mental-physical comorbidity	Mental, behavioural or neurodevelopmental disorders + Symptoms, signs or clinical findings, not elsewhere classified	1	0	3	0	0	1	0	0	5	6.5%
C03	Post-traumatic stress disorder + Substance use disorder	Mental-mental comorbidity	Mental, behavioural or neurodevelopmental disorders + Mental, behavioural or neurodevelopmental disorders	2	0	1	0	0	0	1	0	4	5.2%
C04	Depressive disorder + Post-traumatic stress disorder	Mental-mental comorbidity	Mental, behavioural or neurodevelopmental disorders + Mental, behavioural or neurodevelopmental disorders	2	0	0	1	0	0	0	0	3	3.9%
C05	Chronic pain + Depressive disorder	Mental-physical comorbidity	Mental, behavioural or neurodevelopmental disorders + Symptoms, signs or clinical findings, not elsewhere classified	1	0	0	0	0	0	1	0	2	2.6%
C06	Depressive disorder + Obsessive-compulsive disorder	Mental-mental comorbidity	Mental, behavioural or neurodevelopmental disorders + Mental, behavioural or neurodevelopmental disorders	1	0	0	0	0	1	0	0	2	2.6%
C07	Attention-deficit/hyperactivity disorder + Oppositional defiant disorder	Mental-mental comorbidity	Mental, behavioural or neurodevelopmental disorders + Mental, behavioural or	1	0	0	0	0	0	1	0	2	2.6%

			neurodevelopmental disorders										
C08	COVID-19 + Type 2 diabetes	Physical-physical comorbidity	Endocrine, nutritional or metabolic diseases + Codes for special purposes	0	0	0	0	0	2	0	0	2	2.6%
C09	Depressive disorder + HIV infection	Mental-physical comorbidity	Certain infectious or parasitic diseases + Mental, behavioural or neurodevelopmental disorders	1	0	1	0	0	0	0	0	2	2.6%
C10	Alzheimer disease + Chronic musculoskeletal pain	Physical-physical comorbidity	Diseases of the nervous system + Symptoms, signs or clinical findings, not elsewhere classified	0	0	1	0	0	0	0	0	1	1.3%
C11	Cardiovascular disease + Depressive disorder	Mental-physical comorbidity	Mental, behavioural or neurodevelopmental disorders + Diseases of the circulatory system	1	0	0	0	0	0	0	0	1	1.3%
C12	Ehlers-Danlos syndrome + Type 2 diabetes	Physical-physical comorbidity	Endocrine, nutritional or metabolic diseases + Developmental anomalies	0	0	0	0	0	1	0	0	1	1.3%
C13	Attention-deficit/hyperactivity disorder + Post-traumatic stress disorder	Mental-mental comorbidity	Mental, behavioural or neurodevelopmental disorders + Mental, behavioural or neurodevelopmental disorders	0	0	0	0	0	1	0	0	1	1.3%
C14	Bipolar disorder + Depressive disorder	Mental-mental comorbidity	Mental, behavioural or neurodevelopmental disorders + Mental, behavioural or neurodevelopmental disorders	0	0	1	0	0	0	0	0	1	1.3%
C15	Chronic migraine + Medication-overuse headache	Physical-physical comorbidity	Diseases of the nervous system + Diseases of the nervous system	0	0	0	0	0	0	1	0	1	1.3%
C16	Chronic pain + Early cognitive decline	Physical-physical comorbidity	Symptoms, signs or clinical findings, not elsewhere classified + Symptoms, signs or clinical findings, not	0	0	0	0	0	0	0	1	1	1.3%

			elsewhere classified										
C17	Bipolar disorder + Coronary heart disease	Mental-physical comorbidity	Mental, behavioural or neurodevelopmental disorders + Diseases of the circulatory system	0	0	0	1	0	0	0	0	1	1.3%
C18	Cyclic vomiting syndrome + Superior mesenteric artery syndrome	Physical-physical comorbidity	Diseases of the digestive system + Diseases of the digestive system	0	0	0	0	0	1	0	0	1	1.3%
C19	Depressive disorder + Inflammatory bowel disease	Mental-physical comorbidity	Mental, behavioural or neurodevelopmental disorders + Diseases of the digestive system	1	0	0	0	0	0	0	0	1	1.3%
C20	History of breast cancer + Insomnia disorder	Physical-physical comorbidity	Sleep-wake disorders + Factors influencing health status or contact with health services	0	0	0	0	0	0	1	0	1	1.3%
C21	Meniere disease + Pangastritis	Physical-physical comorbidity	Diseases of the ear or mastoid process + Diseases of the digestive system	0	0	0	0	0	1	0	0	1	1.3%
C22	Alcohol use disorder + Post-traumatic stress disorder	Mental-mental comorbidity	Mental, behavioural or neurodevelopmental disorders + Mental, behavioural or neurodevelopmental disorders	0	0	0	0	0	0	1	0	1	1.3%
C23	Eating disorder + Post-traumatic stress disorder	Mental-mental comorbidity	Mental, behavioural or neurodevelopmental disorders + Mental, behavioural or neurodevelopmental disorders	1	0	0	0	0	0	0	0	1	1.3%
C24	Agoraphobia + Panic disorder	Mental-mental comorbidity	Mental, behavioural or neurodevelopmental disorders + Mental, behavioural or neurodevelopmental disorders	0	0	0	0	0	1	0	0	1	1.3%
C25	Binge-eating disorder + Substance use disorder	Mental-mental comorbidity	Mental, behavioural or neurodevelopmental disorders + Mental, behavioural or	0	0	1	0	0	0	0	0	1	1.3%

			neurodevelopmental disorders										
C26	Depressive disorder + Psychotic disorder	Mental-mental comorbidity	Mental, behavioural or neurodevelopmental disorders + Mental, behavioural or neurodevelopmental disorders	1	0	0	0	0	0	0	0	1	1.3%
C27	Chronic pain + Insomnia disorder	Physical-physical comorbidity	Sleep-wake disorders + Symptoms, signs or clinical findings, not elsewhere classified	0	0	0	0	0	1	0	0	1	1.3%
C28	Migraine + Multiple sclerosis	Physical-physical comorbidity	Diseases of the nervous system + Diseases of the nervous system	1	0	0	0	0	0	0	0	1	1.3%
C29	Anxiety disorder + Persistent postural-perceptual dizziness	Mental-physical comorbidity	Mental, behavioural or neurodevelopmental disorders + Diseases of the ear or mastoid process	1	0	0	0	0	0	0	0	1	1.3%
C30	Depressive disorder + Eating disorder	Mental-mental comorbidity	Mental, behavioural or neurodevelopmental disorders + Mental, behavioural or neurodevelopmental disorders	0	0	0	0	0	1	0	0	1	1.3%
C31	Depressive symptoms + Rheumatoid arthritis	Physical-physical comorbidity	Diseases of the musculoskeletal system or connective tissue + Symptoms, signs or clinical findings, not elsewhere classified	1	0	0	0	0	0	0	0	1	1.3%
C32	Pemphigus vulgaris + Type 2 diabetes	Physical-physical comorbidity	Endocrine, nutritional or metabolic diseases + Diseases of the skin	0	0	0	0	0	1	0	0	1	1.3%
C33	Asthma + Panic disorder	Mental-physical comorbidity	Mental, behavioural or neurodevelopmental disorders + Diseases of the respiratory system	0	0	0	0	0	0	1	0	1	1.3%
C34	Asthma + Vocal cord dysfunction	Physical-physical comorbidity	Diseases of the respiratory system + Diseases of the respiratory system	0	0	0	0	0	1	0	0	1	1.3%
C35	Attention-deficit/hyperactivity	Mental-mental	Mental, behavioural or	0	0	0	0	0	0	1	0	1	1.3%

	disorder + Learning disorder	comorbidity	neurodevelopmental disorders + Mental, behavioural or neurodevelopmental disorders									
C36	Depressive disorder + Hwabyung	Mental-mental comorbidity	Mental, behavioural or neurodevelopmental disorders + Mental, behavioural or neurodevelopmental disorders	1	0	0	0	0	0	0	0	1	1.3%
C37	Generalized anxiety disorder + Hypothyroidism	Mental-physical comorbidity	Endocrine, nutritional or metabolic diseases + Mental, behavioural or neurodevelopmental disorders	0	0	0	0	0	1	0	0	1	1.3%
C38	Depressive symptoms + Insomnia disorder	Physical-physical comorbidity	Sleep-wake disorders + Symptoms, signs or clinical findings, not elsewhere classified	0	0	1	0	0	0	0	0	1	1.3%
C39	Depressive disorder + Internet gaming disorder	Mental-mental comorbidity	Mental, behavioural or neurodevelopmental disorders + Mental, behavioural or neurodevelopmental disorders	0	1	0	0	0	0	0	0	1	1.3%
C40	Post-traumatic stress disorder + Specific phobia	Mental-mental comorbidity	Mental, behavioural or neurodevelopmental disorders + Mental, behavioural or neurodevelopmental disorders	0	0	0	0	0	1	0	0	1	1.3%
C41	Alcohol use disorder + Anxiety disorder	Mental-mental comorbidity	Mental, behavioural or neurodevelopmental disorders + Mental, behavioural or neurodevelopmental disorders	0	0	0	0	0	0	1	0	1	1.3%
C42	Attention-deficit/hyperactivity disorder + Idiopathic epilepsy	Mental-physical comorbidity	Mental, behavioural or neurodevelopmental disorders + Diseases of the nervous system	0	1	0	0	0	0	0	0	1	1.3%

C43	Awake bruxism + Chronic migraine	Physical-physical comorbidity	Diseases of the nervous system + Diseases of the digestive system	1	0	0	0	0	0	0	0	1	1.3%
C44	Borderline personality disorder + Substance use disorder	Mental-mental comorbidity	Mental, behavioural or neurodevelopmental disorders + Mental, behavioural or neurodevelopmental disorders	0	1	0	0	0	0	0	0	1	1.3%
C45	Chronic musculoskeletal pain + Depressive disorder	Mental-physical comorbidity	Mental, behavioural or neurodevelopmental disorders + Symptoms, signs or clinical findings, not elsewhere classified	0	0	0	0	0	0	1	0	1	1.3%
C46	Anxiety disorder + Depressive disorder	Mental-mental comorbidity	Mental, behavioural or neurodevelopmental disorders + Mental, behavioural or neurodevelopmental disorders	1	0	0	0	0	0	0	0	1	1.3%
C47	Major depressive disorder + Obsessive-compulsive disorder	Mental-mental comorbidity	Mental, behavioural or neurodevelopmental disorders + Mental, behavioural or neurodevelopmental disorders	0	0	1	0	0	0	0	0	1	1.3%
C48	Panic disorder + Personality disorder	Mental-mental comorbidity	Mental, behavioural or neurodevelopmental disorders + Mental, behavioural or neurodevelopmental disorders	1	0	0	0	0	0	0	0	1	1.3%
C49	Irritable bowel syndrome + Post-traumatic stress disorder	Mental-physical comorbidity	Mental, behavioural or neurodevelopmental disorders + Diseases of the digestive system	0	0	1	0	0	0	0	0	1	1.3%
C50	Mild traumatic brain injury + Post-traumatic stress disorder	Mental-physical comorbidity	Mental, behavioural or neurodevelopmental disorders + Injury, poisoning or certain other consequences of external causes	0	0	0	0	0	0	1	0	1	1.3%

C51	Hypertension + Type 2 diabetes	Physical-physical comorbidity	Endocrine, nutritional or metabolic diseases + Diseases of the circulatory system	0	0	0	0	1	0	0	0	1	1.3%
C52	Depressive disorder + Drug dependence	Mental-mental comorbidity	Mental, behavioural or neurodevelopmental disorders + Mental, behavioural or neurodevelopmental disorders	1	0	0	0	0	0	0	0	1	1.3%
C53	Bipolar disorder + Post-traumatic stress disorder	Mental-mental comorbidity	Mental, behavioural or neurodevelopmental disorders + Mental, behavioural or neurodevelopmental disorders	1	0	0	0	0	0	0	0	1	1.3%

Note: RCT = randomised controlled trial; NRCT = non-randomised controlled trial. Condition pairs are nondirectional. WHO ICD-11 source: https://icd.who.int/browse/2026-01/mms/en

Table S6. Study-level distribution of outcome domains and directions

Counts represent unique studies; percentages use all 77 clinical studies as the denominator.

Outcome domain	Assessed		Beneficial		Non-significant		Unfavourable		Favourable safety		Safety concerns	
	n	%	n	%	n	%	n	%	n	%	n	%
Psychological outcomes	65	84.40%	56	72.70%	36	46.80%	8	10.40%	0	0.00%	0	0.00%
Clinical symptoms	48	62.30%	33	42.90%	23	29.90%	5	6.50%	12	15.60%	2	2.60%
Healthcare utilisation and economic outcomes	39	50.60%	29	37.70%	7	9.10%	17	22.10%	0	0.00%	0	0.00%
Physiological or biomarker outcomes	27	35.10%	23	29.90%	13	16.90%	3	3.90%	0	0.00%	0	0.00%
Physical functioning	16	20.80%	11	14.30%	6	7.80%	0	0.00%	0	0.00%	0	0.00%
Health-related quality of life	15	19.50%	10	13.00%	10	13.00%	2	2.60%	0	0.00%	0	0.00%
Subjective well-being	13	16.90%	10	13.00%	3	3.90%	1	1.30%	0	0.00%	0	0.00%
Sleep	11	14.30%	8	10.40%	4	5.20%	0	0.00%	0	0.00%	0	0.00%
Social relationships	8	10.40%	6	7.80%	2	2.60%	0	0.00%	0	0.00%	0	0.00%

Overall outcome directions

Direction	Studies n	Studies %
Beneficial	75	97.40%
Non-significant	51	66.20%
Unfavourable	28	36.40%
Favourable safety findings	12	15.60%
Safety concerns	2	2.60%

Notes: Outcome domains and directions were not mutually exclusive; a study could contribute to more than one domain and direction. These frequencies are descriptive and should not be interpreted as pooled estimates of efficacy or safety.
